# A New Strategy Based on LC-Q TRAP-MS for Determining the Distribution of Polyphenols in Different Apple Varieties

**DOI:** 10.3390/foods11213390

**Published:** 2022-10-27

**Authors:** Minyan Wang, Zhangzhen Bai, Huili Zhu, Tiantian Zheng, Xiujiao Chen, Pengmin Li, Jing Zhang, Fengwang Ma

**Affiliations:** 1 State Key Laboratory of Crop Stress Biology for Arid Areas/Shaanxi Key Laboratory of Apple, College of Horticulture, Northwest A&F University, Xianyang 712100, China; 2 College of Landscape Architecture and Arts, Northwest A&F University, Xianyang 712100, China

**Keywords:** polyphenols, apple, spatial distribution pattern, liquid chromatography-quadrupole ion trap-mass spectrometry (LC-Q TRAP-MS), multiple reaction monitoring

## Abstract

Apples are a rich source of polyphenols in the human diet. However, the distribution of polyphenols in different apple varieties and tissues is still largely unclear. In this study, a new liquid chromatography–tandem mass spectrometry (LC-MS/MS) strategy was developed to reveal the spatial distribution of polyphenols in different apple tissues and varieties. A method based on multiple reaction monitoring (MRM)-enhanced product ion (EPI) was established in the information-dependent acquisition (IDA) mode for pseudo-target screening of major apple polyphenols. A total of 39 apple polyphenolic metabolites were finally identified. Qualitative and quantitative results showed that the variety and content of polyphenols in apple peels were higher than those of other tissues. In apple roots, stems, and leaves, the highest polyphenol variety and content were found in wild species, followed by cultivars and elite varieties. Dihydrochalcone substances, one kind of major apple polyphenols, were more abundant in apple roots, stems, and leaves. This strategy can be applied as a model for other agricultural products, in addition to revealing the distribution of polyphenols in different tissues of apples, which provides a theoretical basis for the utilization of polyphenol resources and variety selection.

## 1. Introduction

Polyphenols are beneficial to human health and are found in a variety of fruits, foods, and beverages. Apples are a major source of polyphenols in the human diet [1,2]. Many studies report that polyphenols can prevent coronary heart disease and cancer [3], obesity, and high cholesterol [4], reduce inflammation [5], and act as antioxidants [6]. Polyphenols are widely used in cosmetics, pharmaceuticals, and food because of their functional versatility [7]. In addition, higher plants produce polyphenols that can protect them from biotic and abiotic stresses [8]. Polyphenols, as part of plant defense, exist as glycosides, acyl sugars, and other conjugated forms rather than glycosides; biotic or abiotic stress can inactivate its enzymatic activity by hydrolyzing glycosides to release more active aglycones, protecting against stress [9].

Phenolic compounds are present in plants in the form of glycosides or esterified with carboxylic acids, with apples having the highest free phenolic content [10]. Phenolic acids and flavonoids are the major polyphenolic substances in apples [11], and flavonoids are subdivided into flavonols, flavanols, dihydrochalcone, and anthocyanins [12]. There are differences in the species and content of apple polyphenols in different varieties and tissues [13]. However, most of the current research on apple polyphenols focuses on flesh and peel, for example, Bai [14], Mari [15], and Barbara [16]. The distribution of polyphenols in roots, stems, and leaves has been less studied. Therefore, it is crucial to systematically explore the distribution and content of polyphenols in different tissues and varieties of apples to expand the utilization of apple polyphenol resources, evaluate their potential beneficial health value, and select excellent varieties.

The total amount of metabolites in plants is huge, and liquid chromatography coupled with tandem mass spectrometry (LC-MS/MS) has the advantages of high sensitivity, good selectivity, high throughput, good reproducibility, and wide coverage of metabolites, which is essential for the characterization and quantification of metabolites and has become an indispensable analytical technique for the study of metabolomics [17,18]. In targeted metabolomics, a triple quad mass spectrometer can be used with high sensitivity, good reproducibility, and a wide linear dynamic range in MRM mode [19]. However, this method is only for metabolites with chemical standards. Non-targeted methods typically detect as many metabolites as possible without knowing the composition of the sample [20,21]. However, there are still problems with complex matrices [22], peak alignment defaults [23], and narrow linear ranges in large-scale non-targeted measurements [24]. Therefore, the accurate identification and quantification of unknown or known metabolites remain a challenge in the absence of available chemical standards.

In this study, we developed a systematic workflow for the characterization and quantification of polyphenol metabolites in apples using an LC-Q TRAP-MS strategy. The rapid, facile simultaneous measurement of multiple polyphenols under conditions that do not rely on chemical standards and allow for the purposeful selection of ions within a narrow scan range using MRM mode to improve the stable linearity of metabolites. To systematically analyze the distribution of polyphenols in different tissues of apples and to quantify the content of polyphenols in different varieties, the utilization of polyphenol resources was expanded. The method can be used as a model strategy for the analysis of polyphenolic substances in different types of plant materials.

## 2. Materials and Methods

### 2.1. Chemicals

All the used reagents and solvents, including High Performance Liquid Chromatography (HPLC)-grade methanol, acetonitrile, and formic acid, were purchased from Merck (Darmstadt, Germany). Ultrapure water using Milli-Q water purification system (Millipore, Bedford, MA, USA).

### 2.2. Plant Materials

Different plant materials of apples, including roots, stems, leaves, peels, and flesh, were obtained from the Horticultural Experimental Station of Northwest A&F University, Yangling, China (108°24′ E, 34°20′ N). Roots, stems, and leaves were collected during the early plant growth period, from three varieties of *Malus sieversii* (MS), *Malus hupehensis* (MH), and Gala (GL), respectively. Apple flesh and peel from elite varieties, cultivars, and *Malus prunifolia* (MP), were harvested at maturity, apple flesh more exactly: 10-23, X94-134, 2-14, X87-65, 63-95, N49-7, CF, GL, Fuji (FJ), Qincui (QC), Qinguan (QG), Honey Sweet (HS), and Mardi (MD), apple peels more exactly: X90-30, L31-67, X83-92, 2-14, 71-31, 67-63, 63-95, 52-46, FJ, QC, QG, HS, and MD. After all samples were cleaned, the apple peel was taken evenly with a peeler, the apple peel from different parts was mixed, frozen in liquid nitrogen, and then ground to a powder using a liquid nitrogen grinder (A11, Null, Germany) and placed at −80 °C before further use. Three biological replicates were set up, with 8 fruits per replicate.

### 2.3. Sample Preparation

An amount of 0.5 g of apple powder was weighed three times, including apple roots, stems, leaves, peels, and flesh, and 1.0 mL of 75% acidified methanol was added, followed by ultrasonic (KQ-100DE, Kunshan ultrasonic instrument co., ltd, Kunshan, China) extraction for 30 min at 20 °C. After centrifugation (AG22331, Eppendorfa, Germany) at 10,000× *g* for 10 min, the supernatant was aspirated. The filtrate was then filtered using 0.22 μm membrane, packed into brown amber vials, and stored at −80 °C until LC-MS analysis. An amount of 20 μL of each extract was mixed to prepare a quality control (QC) sample that was used to evaluate the reproducibility and robustness of the proposed non-targeted LC-MS/MS analytical method.

### 2.4. HPLC Conditions

Sample extracts were analyzed using an liquid chromatography-electrospray ionization-tandem mass spectrometry (LC-ESI-MS/MS) system (HPLC, Shim-pack UFLC SHIMADZU CBM20A system; MS, Applied Biosystems 5500 Q TRAP). The analytical conditions were as follows, HPLC: InerSustain AQ-C18 (150 mm × 4.6 mm, 5 μm, Shimadzu, Kyoto, Japan) chromatography columns; solvent system: A (0.1% formic acid in ultrapure water) and solvent B (acetonitrile), and the flow rate was set to 0.4 mL/min. The elution gradient pattern was as follows: 10% B at 0–0.01 min; 10–50% B at 0.01–7 min; 50–65% B at 7–9 min; 65–85% B at 9–12 min; 85–95% B at 12–13 min 95% B at 13–16.5 min; 95–10% B at 16.5–16.6 min; 10% B at 16.6–20 min. The column oven temperature was 40 °C. The sample injection volume was 2.0 μL. The analyte effluent connected to an ESI-triple quadrupole-linear ion trap (Q TRAP)-MS.

### 2.5. ESI-Q TRAP-MS/MS

Mass spectrometry was performed using an AB SCIEX Q TRAP 5500 (Applied Biosystems, Foster, CA, USA)and a turbo electrospray ionization (ESI) source to obtain linear ion trap (LIT) and triple quadrupole (QQQ) scans. Experiments were set up in positive and negative ion modes. The ESI source in negative ion mode operates as follows: ion spray voltage, temperature, ion source GasI (GSI), GasII (GSII), and curtain gas were set at −4500 V, 600 °C, 60, and 30 psi, respectively; collision gas was set high. The ESI source in positive ion mode was the same as the negative ion mode except that the IS was 5500 V.

### 2.6. LIT Experiments

Multiple ion monitoring (MIM) scans were performed as survey scans to trigger IDA of the EPI scan model MIM-EPI to screen metabolites [25]. Uesd the same ion pair (Q1–Q3). In negative ion mode, declustering potential (DP) was set as −60 V, and collision energy (CE) was set as −30 V with a tolerance of 15. In positive ion mode, DP was set as 60 V, and CE was set as 30 V with a tolerance of 15. The mass range was from *m*/*z* 50 to 700. MS data were obtained by the IDA method, and 2–3 characteristic ion peaks were selected.

### 2.7. QQQ Experiments

In the LIT experiments, after selecting 2–3 characteristic ion peaks, the characteristic ion pairs (Q1–Q3) were entered manually. Quantification of apple polyphenols was obtained by QQQ scanning in MRM mode. The characteristic ion pairs (Q1–Q3) were selected and flow injection analysis (FIA) was automatically optimized in different positive and negative ion modes to obtain the optimum DP and CE. Different ranges were set for DP and CE in positive and negative ion modes, respectively, to obtain optimal values. After optimization, a targeted quantitative approach was established [26]. Additionally, IDA-triggered EPI scans were performed to confirm targeting of apple polyphenolic compounds, which were same conditions as those obtained for product ion analysis under MIM conditions. Date were processed using Analyst 1.6.3 software; peak areas were processed using MultiQuant 3.0.2 software (AB SCIEX, Foster City, CA, USA).

### 2.8. Data Processing and Statistical Analysis

Data were shown as the mean ± standard deviation of three independent measurements, calculated using Microsoft Excel 2010(Microsoft, Washington, DC, USA). Using IBM SPSS 19.0 software (Armonk, NY, USA), the significance of the data was analyzed by one-way ANOVA at *p* < 0.05 level. Heat maps, principal component analysis (PCA), stacked maps, data processing, *t*-tests, and data visualization were all processed using the R language (version 4.2.2, Vienna, Austria).

## 3. Results and Discussion

### 3.1. Optimization of Chromatographic Conditions for LC-Q TRAP-MS

**Figure 1:**
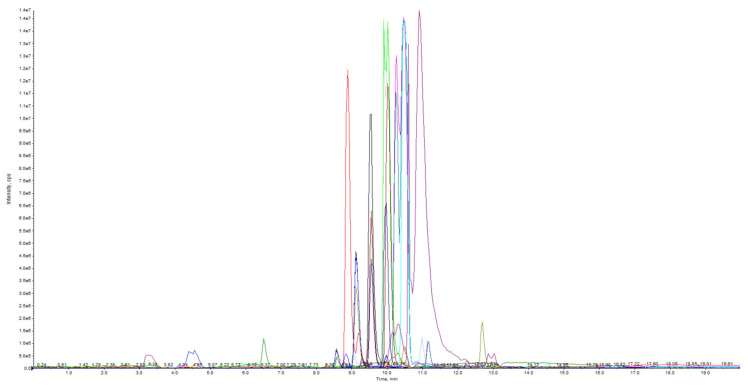
HPLC-MS/MS chromatograms of 39 polyphenol metabolites in apple in multiple reaction monitoring (MRM) mode.

Chromatographic conditions, including column, column temperature, mobile phase, flow rate, and gradient elution, have a great influence on the separation efficiency of metabolites [26]. A mixed sample QC containing all metabolites of apple polyphenols was prepared prior to the assay. Compared with the Kinetex F5 column (3.0 mm × 100 mm, 2.6 μm) (Phenomenex, Los Angeles, CA, USA), the AQ-C18 column (4.6 mm × 150 mm, 5 μm, Shimadzu Corp., Tokyo, Japan) showed better resolution and peak shape on apple polyphenols. After that, the column temperature and flow rate were tested separately, showing that 40 °C temperature and 0.4 mL/min flow rate were better than other conditions. The mobile phases were better separated by phase A (0.1% formic acid in ultrapure water) and phase B (acetonitrile), respectively [27]. In order to improve the separation efficiency of apple polyphenols and shorten the detection time, the linear gradient elution process was optimized by adjusting the volume ratio of phase A and phase B in combination with the MIM map separation debugged several times. The final optimized chromatographic conditions for HPLC were described in Section 2.4. The base peak chromatogram (BPC) of QC samples obtained from MRM-IDA-EPI analysis further demonstrates the chromatographic separation of apple polyphenols (Figure 1). The different colors in Figure 1 were the mass spectra with different *m*/*z* values captured, representing the different polyphenolic substances in apples.

### 3.2. Identification of Apple Polyphenol Components and MS^2^ Spectral Tag (MS2T) Library Construction

**Figure 2:**
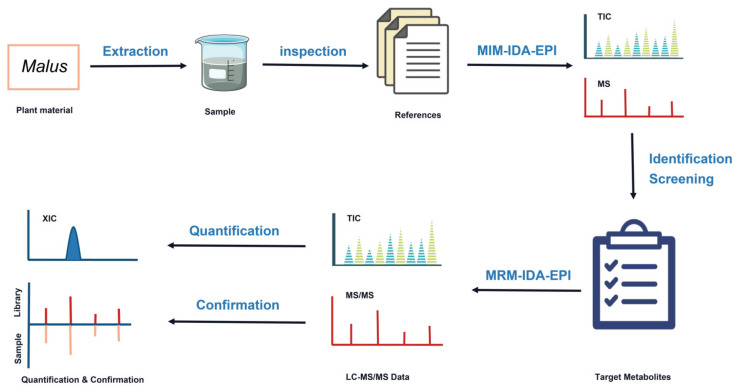
Workflow based on integrated strategy of HPLC-Q TRAP-MS/MS for revealing the spatial distribution patterns of apple polyphenol metabolites.

Previously, the major polyphenol metabolites in apples were searched from a large number of literature, and the Q1 *m*/*z* of each polyphenol compound was precisely obtained. An MIM-EPI method was used to study the polyphenolic substances of apple species by HPLC-Q TRAP-MS/MS combined with the IDA method in a highly sensitive and rapid manner [25,28]. The main processes are shown in Figure 2, and details of the process are described in Section 2.

By using the MIM-EPI method, a total of less than 1000 MS2 spectral signals were obtained from 70 MIMs in positive and negative ion modes, respectively. For each metabolite pattern fragment obtained by ESI-Q TRAP-MS/MS, the primary peaks of the secondary profile were recorded, compared with standard compound, literature search, and database search, and retention times were recorded. The MS2T library was annotated according to the fragmentation pattern, retention time, and exact *m*/*z* value of each metabolite. A total of 39 apple polyphenol metabolites were identified. The construction of a high-quality library containing 39 apple polyphenols facilitated targeted LC-MS methods for the simultaneous identification and quantification of polyphenolic substances in apples. The method was improved by introducing a stepwise MIM-EPI-based strategy for the construction of an MS2T library. Executed in LIT mode, accumulation of fragment ions in Q TRAP instrument Q3 for increased sensitivity in EPI scans [29]. Therefore, the stepwise MIM-EPI method solves the need for standards in traditional methods and detects as many polyphenols as possible.

### 3.3. Targeted Quantification and Validation of Apple Polyphenols

At least one diagnostic ion of each target compound was screened by the established MS2T library for MRM analysis. In order to generate the maximum signal, DP and CE optimization is usually performed for each premise-product ion (Q1–Q3) transition. For metabolites without chemical standards, we used FIA, a method that is independent of the chemical standard. In the scheduled MRM mode, DP and CE parameters can be automatically optimized in different positive and negative ion modes using the identified endogenous metabolites. The optimization results were shown in Table 1, and the MS2 spectra of 39 polyphenol metabolites in apples were shown in Figure S1. To avoid false-positive results due to the number of ion pairs, IDA-triggered EPI scans were performed on the MS2 library to confirm the metabolites. This total integrated MRM-IDA-EPI approach has the advantage of simultaneous identification and quantification of target metabolites [26].

To verify the reproducibility of the method, the analysis of apple polyphenol substances in QC samples was repeated. The coefficient of variation (CV) of the retention time (RT) was less than 0.95% and the CV of peak areas for all targeted metabolites was below 4.92% (Table S1). The data showed that the quantitative method has good repeatability, stability, accuracy, and precision.

### 3.4. Distribution of Polyphenolic Compounds in Different Varieties and Tissues of Apple

**Figure 3:**
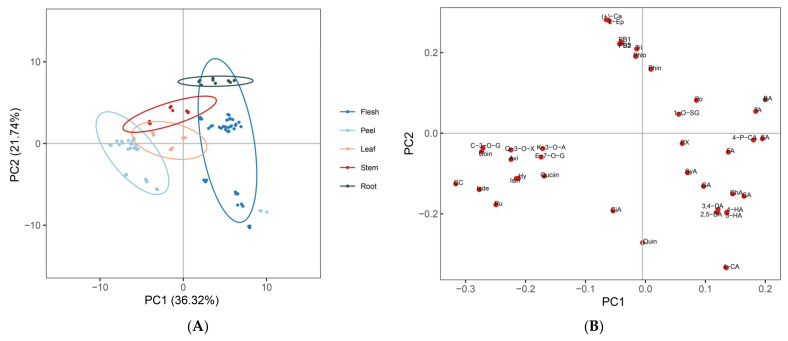
Score plot (**A**) and loading plot (**B**) of principal component analysis polyphenol metabolites in different tissues of apple. Individual letter abbreviations: 4-P-CA: 4-*P*-coumaroylquinic acid; (+)-Ca: (+)-Catechin; 1-*O*-SG: 1-*O*-Sinapoyl-β-D-glucose; 2,5-DA: 2,5-Dihydroxybenzoic acid; 3,4-DA: 3,4-Dihydroxybenzoic acid; 4-CA: 4-caffeoylquinic acid; 4-HA: 4-Hydroxycinnamic acid; Avi: avicularin; BA: Benzoic acid; CA: Caffeic acid; ChA: Chlorogenic acid; CiA: cinnamic acid; FA: Ferulic acid; GA: Gallic acid; Hy: Hyperoside; Isin: Isoquercitrin; Isde: Isoquercitroside; K-3-O-A: Kaempferol 3-*O*-arabinoside; L-Ep: L-Epicatechin; E-7-O-G: Eriodictyol-7-*O*-glucoside; PX: phloretin xyloglucoside; Phin: phlorizin; Po: Polydatin; PB1: Procyanidin B1; PB5: Procyanidin B5; PB2: Procyanidin B2; Quin: Quercetin; Q-3-O-X: Quercetin 3-*O*-β-D-xylopyranoside; Quciin: Quercitrin; SA: Salicylic acid; SyA: syringic acid; TA: Terephthalic acid; 3-HA: 3-Hydroxycinnamic acid; Phlo: Phloretin; Coin: Cosmosiin; C-3-O-G: Cyanidin 3-*O*-glucoside; Tri: trilobatin; CC: Cyanidin Chloride; Ru: Rutin.

Based on our established new method, 39 polyphenolic substances in apple roots, stems, leaves, peels, and flesh were successfully quantified and identified. The PCA was performed to investigate the distribution relationships of apple polyphenol metabolites among different tissues of different varieties (Figure 3). The PCA score plot (PC1: 36.32%, PC2: 21.74%) showed the separation of 39 polyphenol metabolites in different tissues of different apple varieties (Figure 3A). In apple stems, GL was opposite to MS and MH in terms of PC2 composition, while in leaves, GL was opposite to MS and MH in terms of PC1 composition, indicating the types and contents of polyphenol metabolites in cultivars and wild species differed greatly. Polyphenol aggregation in apples is mainly divided into three categories: flavanols and dihydrochalcones; phenolic acids; flavonols and anthocyanins. (Figure 3B).

**Figure 4:**
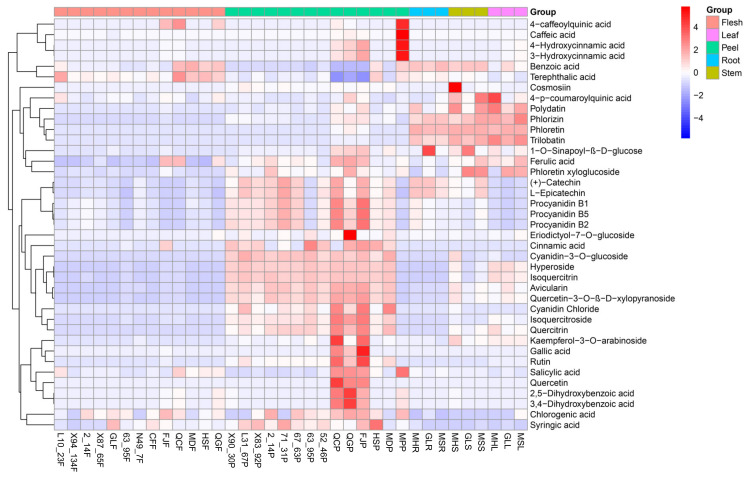
Heatmap of polyphenol metabolites in different tissues of apple.

For quantitative comparison, we summarized the content of apple polyphenols in different tissues of different varieties (Figure 4). The results showed that the contents and species of apple peel polyphenols in cultivated species were higher than those in wild species and elite varieties, suggesting that apple polyphenols were enhanced during domestication to improve susceptibility/tolerance levels to fungal infections and insects [30,31,32]. Among the different tissues of apples, the species and content of polyphenols in the peel were higher than those in other tissues of apples, due to the fact that the peel is an important source of antioxidants with higher content of phenolic compounds, antioxidant activity, and antiproliferative activity [33]. In different tissues of different apples, especially in peels, roots, stems, and leaves, the species and contents of polyphenols showed significant differences, suggesting that phenolic compound biosynthesis is tissue-specifically regulated and has an important role in this regulatory mechanism’s genotype-specific characteristics [34]. It is noteworthy that three cultivars, QC, QG, and FJ, had higher contents of gallic acid, rutin, salicylic acid, quercetin, 2,5-dihydroxybenzoic acid, and 3,4-dihydroxybenzoic acid in the peel than other elite varieties, which indicates that the cultivars contain more polyphenolic substances after domestication. To our knowledge, cosmosiin was first found in apples, and MH stems contain large amounts of cosmosiin.

**Figure 5:**
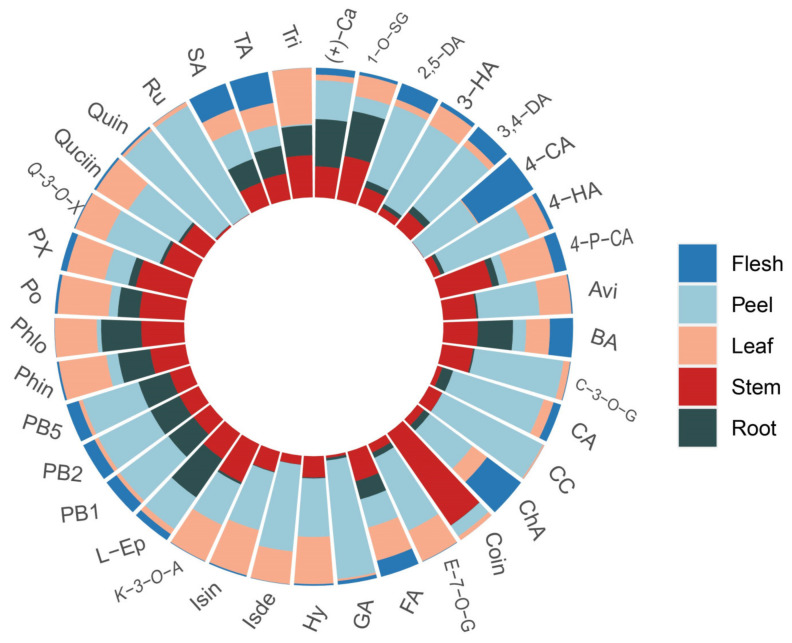
Percentage of 39 polyphenolic metabolites in different tissue species of apple.

To explore the proportion of 39 polyphenols in different tissues of apples, the analysis was shown in Figure 5. The results showed that the distribution of polyphenol metabolites in different tissues was different. Caffeic acid (73.40%), cyanidin chloride (87.24%), and cyanidin 3-*O*-glucoside (69.18%) were mainly found in apple peels because of their protective effect against biotic and abiotic stresses, especially their astringent properties that protect the fruit from pathogens and predators [35,36]. Additionally, rutin (96.05%) and quercetin (94.19%) were mainly present in the apple peel. Chlorogenic acid is an effective antioxidant, and the inhibition of lignin synthesis can prevent the formation of yellow–brown apple fruit and inhibit postharvest decay [37,38,39]. In this experiment, chlorogenic acid was mainly distributed in apple peel and flesh, which could enhance the antioxidant properties of the fruit. Dihydrochalcones are unique polyphenols of the apple genus [40]. In this experiment, dihydrochalcones (phloretin xyloglucoside 76.67%, phlorizin 89.51%, phloretin 96.24, trilobatin 98.91%) mainly existed in apple roots, stems, and leaves. The proportion of phloretin in apple roots, stems, and leaves was higher than that of phloretin, which may be due to the partial hydrolysis of glycosides to aglycones during growth [35]. These polyphenol metabolites showed different proportions in different tissues, indicating that apple polyphenols are different in different tissues [41]. The results of this study showed that the varieties and contents of polyphenols in different tissues were different, and they could be extracted according to needs, which expanded the utilization range of apple polyphenols.

## 4. Conclusions

In IDA mode, 39 polyphenolic substances were identified from apple roots, stems, leaves, peels, and flesh by HPLC-Q TRAP-MS/MS, and in MRM mode, polyphenolic substances in different tissues of different varieties were quantified by MRM-IDA-EPI mode. The results revealed the spatial distribution pattern of polyphenolic substances in different tissues of apples and quantified the polyphenolic substances in different varieties. According to the results, the types and contents of polyphenols in apple peel were higher than those in other tissues, and dihydrochalcone substances mainly existed in the roots, stems, and leaves of apples. Among different varieties, the types and contents of polyphenols in apple fruit of cultivated varieties were higher than those of elite varieties, and the polyphenols in roots, stems, and leaves of wild varieties were higher than those of cultivated varieties. To the best of our knowledge, cosmosiin was first found in apples, and MH stems contain large amounts of cosmosiin. These results may expand the utilization of apple polyphenol resources and also provide a theoretical basis for the selection of elite varieties.

## Figures and Tables

**Table 1:** A list of 39 apple polyphenol metabolites.

Metabolite Name	RT (min)	Q1	Q3	DP	CE	Metabolite Name	RT (min)	Q1	Q3	DP	CE
4-*P*-coumaroylquinic acid	9.46	337	173	−50	−20	Caffeic acid	10.14	179	134.9	−73.13	−21
1-*O*-Sinapoyl-β-D-glucose	8.62	385	223	−90	−20	Chlorogenic acid	8.95	353	191	−64	−22
3,4-Dihydroxybenzoic acid	9.03	153	109	−30	−20	Cinnamic acid	6.53	147	103	−20	−15
4-caffeoylquinic acid	4.10	353	173	−50	−20	Ferulic acid	11.35	193	148.9	−67.52	−20.33
4-Hydroxycinnamic acid	11.08	163	119	−60	−20	Gallic acid	11.47	169	125	−27	−19
Avicularin	10.46	433	300	−60	−35	Hyperoside	10.06	463	300	−48	−37
Benzoic acid	9.84	121.9	92	−52.02	−16.06	Phlorizin	10.61	435	273	−175	−23
Isoquercitrin	10.00	463	300	−20	−35	Polydatin	10.13	389	227	−188	−20
Isoquercitroside	9.97	463.38	271	−90	−39	Procyanidin B1	9.67	577	407	−50	−30
Kaempferol 3-*O*-arabinoside	10.92	417	285	−90	−25	procyanidin B5	9.22	577	289	−70	−35
L-Epicatechin	9.58	289	245	−125.1	−25.79	Procyanidin B2	9.07	577	289	−60	−35
Eriodictyol-7-*O*-glucoside	11.07	449	287	−60	−50	Terephthalic acid	3.32	165	121	−90	−15
Phloretin xyloglucoside	10.77	567	273	−40	−35	3−Hydroxycinnamic acid	11.08	163	119	−40	−20
Quercetin	12.62	301	151	−123	−28	Phloretin	13.08	275	169	20	20
Quercetin 3-*O*-β-D-xylopyranoside	10.48	433	301	−90	−30	Cosmosiin	8.39	433	271	60	25
Quercitrin	10.49	447	300	−90	−35	Cyanidin 3-*O*-glucoside	8.29	449	287	10	27
Salicylic acid	10.15	137.3	92.8	−99.31	−25.85	Rutin	9.51	611	303	80	25
Syringic acid	6.24	197.9	153	−53.16	−17.29	Trilobatin	10.67	437.3	275.1	80	15
(+)-Catechin	9.58	289	203	−35.99	−26.8	Cyanidin Chloride	9.63	287	213	115	43
2,5-Dihydroxybenzoic acid	9.07	153	109	−67.97	−27.9						

## Data Availability

Data are contained within the article or Supplementary Materials.

## Supplementary Materials

The following supporting information can be downloaded at: https://www.mdpi.com/article/10.3390/foods11213390/s1, Figure S1: MS2 spectrometry of 39 polyphenol metabolites in apple; Table S1: Coefficient of variation (CV) of 39 apple polyphenol metabolites.
