# Supplementary material for: A New Strategy Based on LC-Q TRAP-MS for Determining the Distribution of Polyphenols in Different Apple Varieties
Source: Foods. 2022 Oct 27;11(21):3390. doi: 10.3390/foods11213390 (PMC9657627; doi:10.3390/foods11213390)
Supplement: Supplementary file 1 [file foods-11-03390-s001.zip › Figure S1.pdf]

1-O-Sinapoyl-β-D-glucose

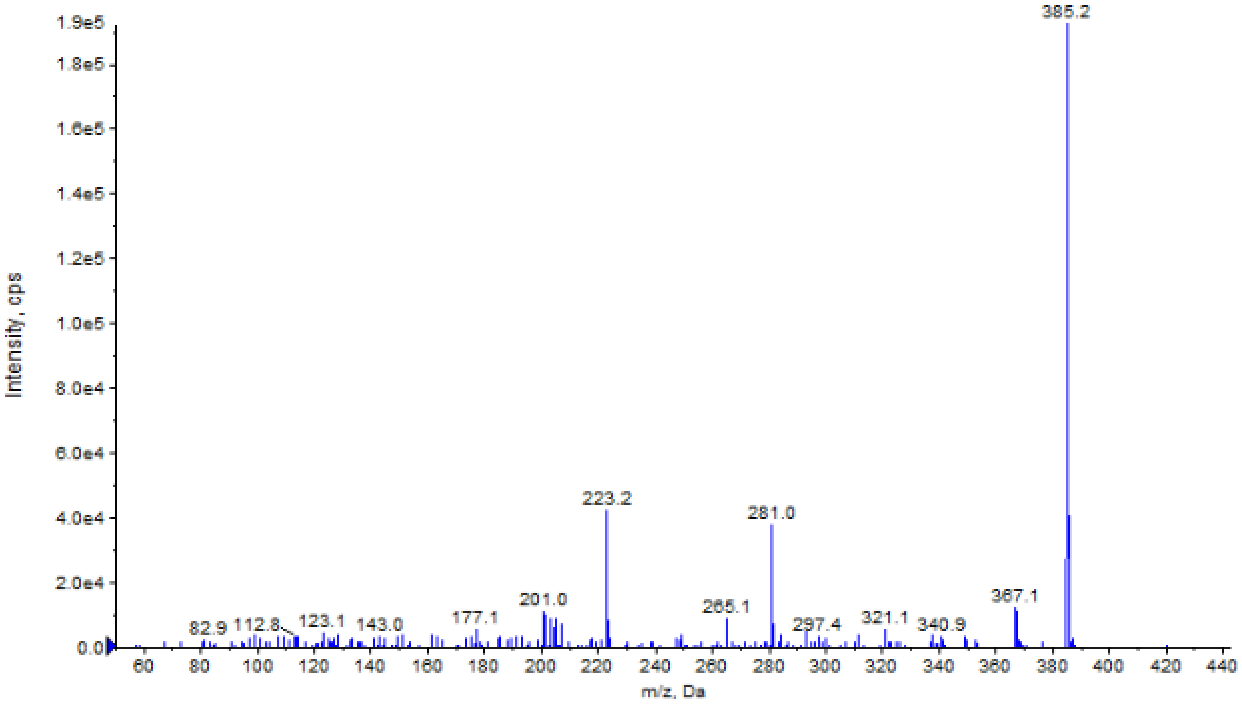

4-P-coumaroylquinic acid

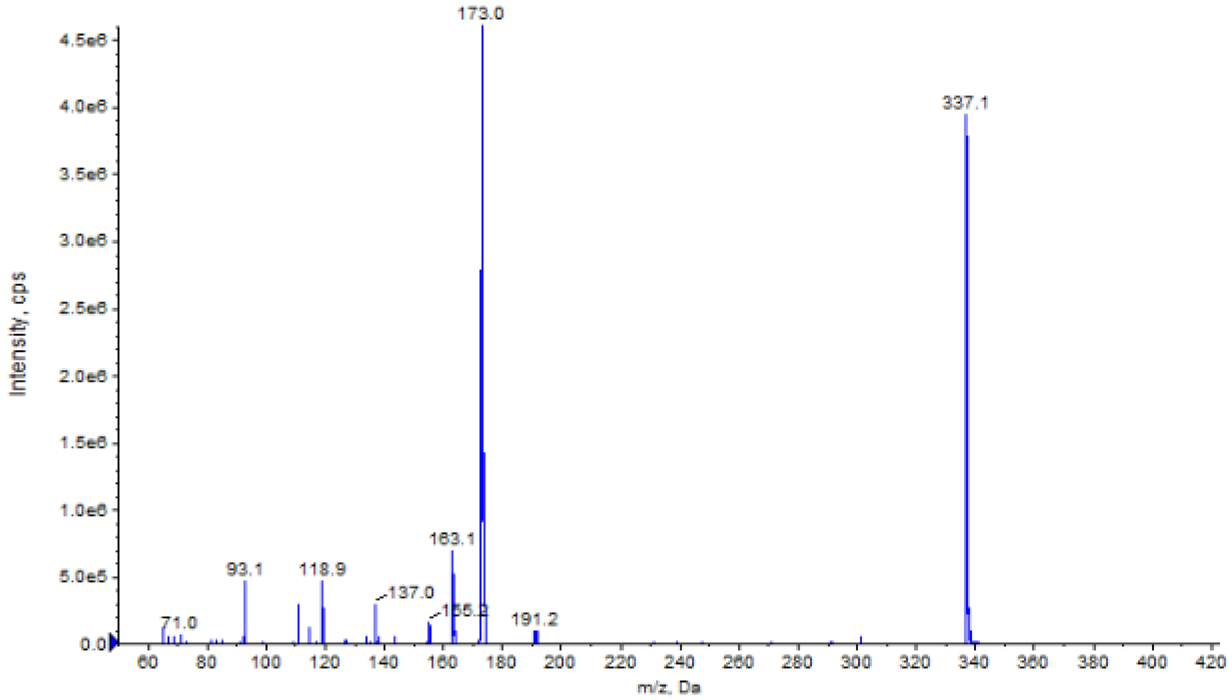

3,4-Dihydroxybenzoic acid

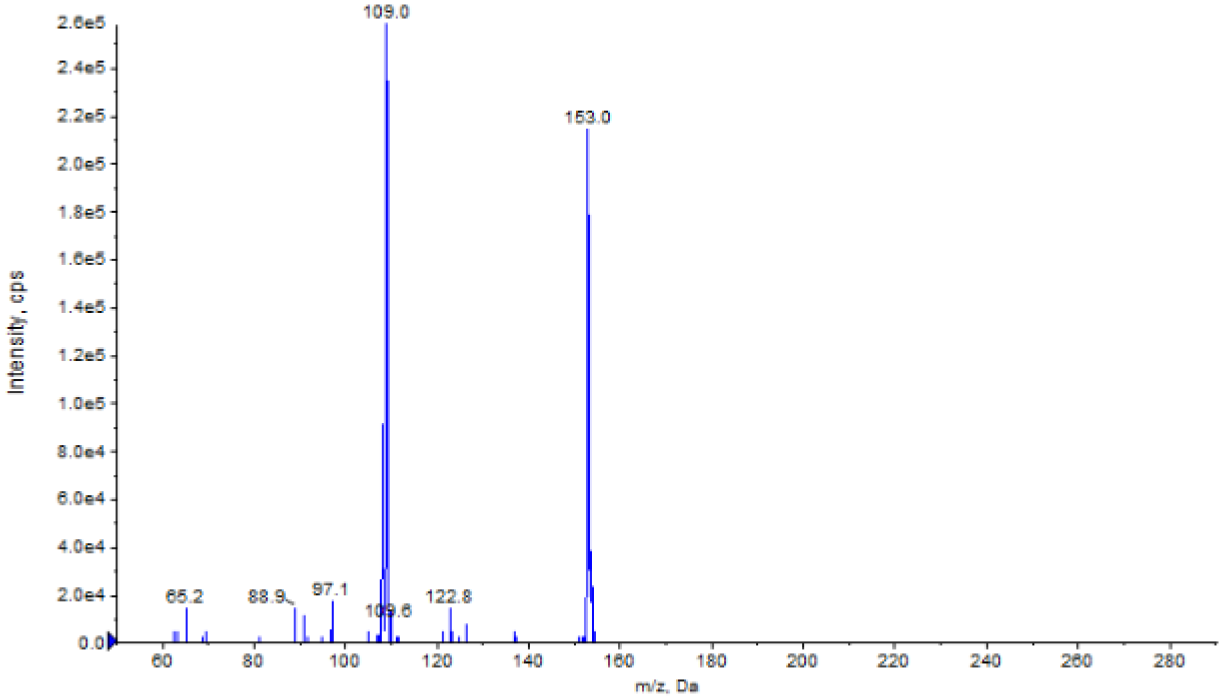

4-caffeoylquinic acid

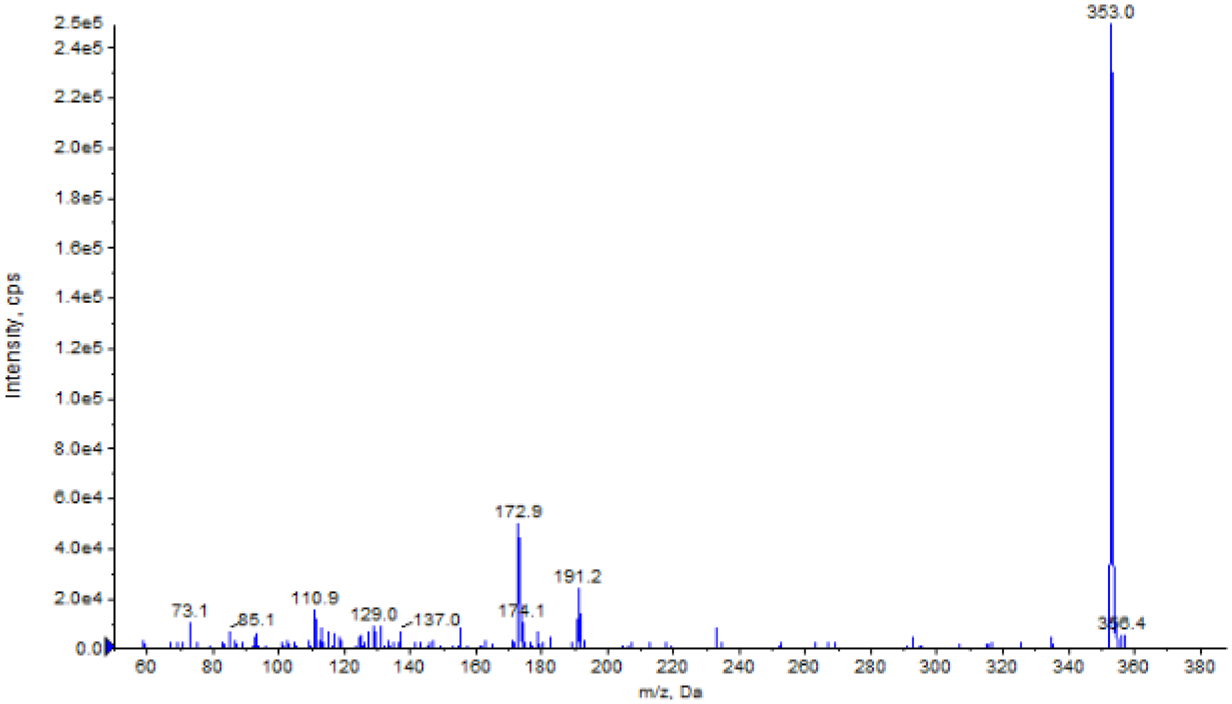

4-Hydroxycinnamic acid

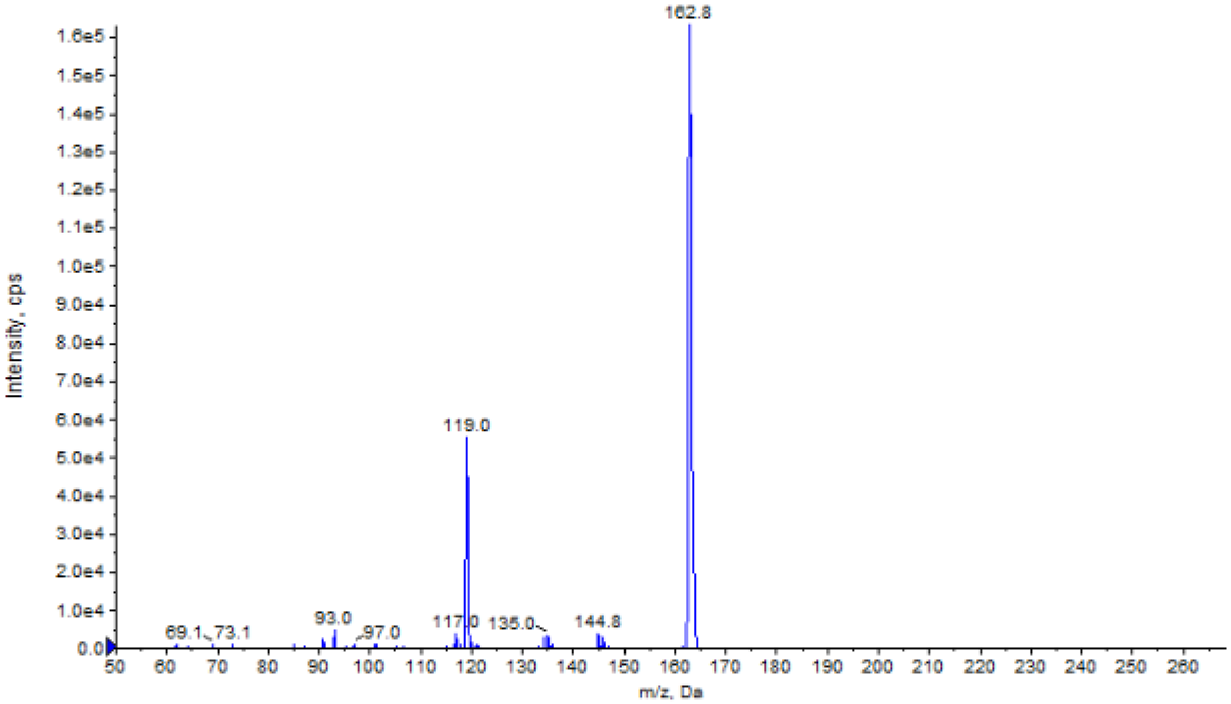

Avicularin

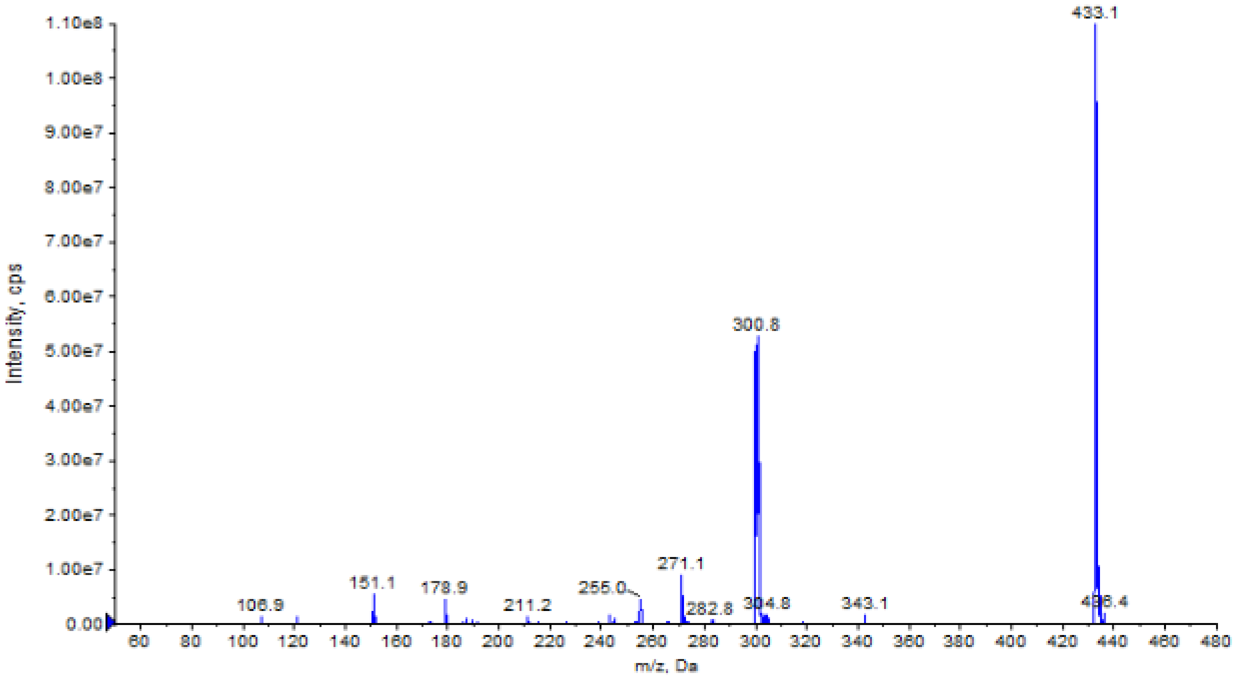

Benzoic acid

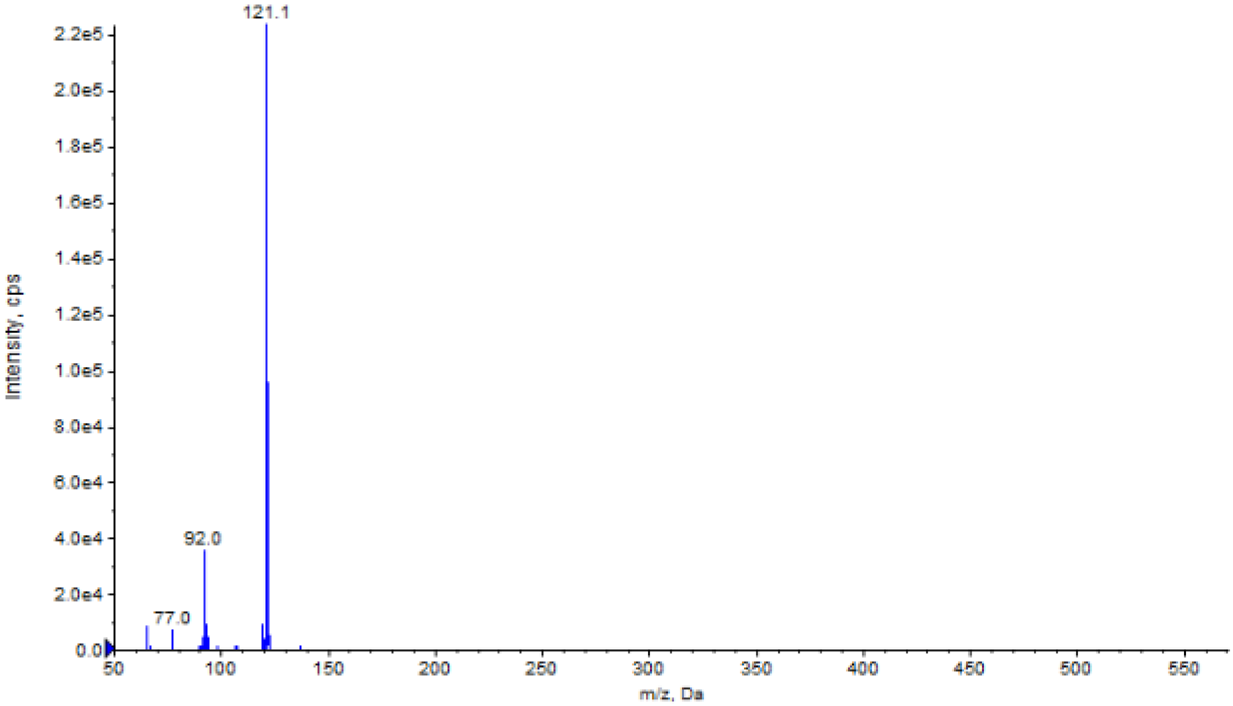

Isoquercitrin

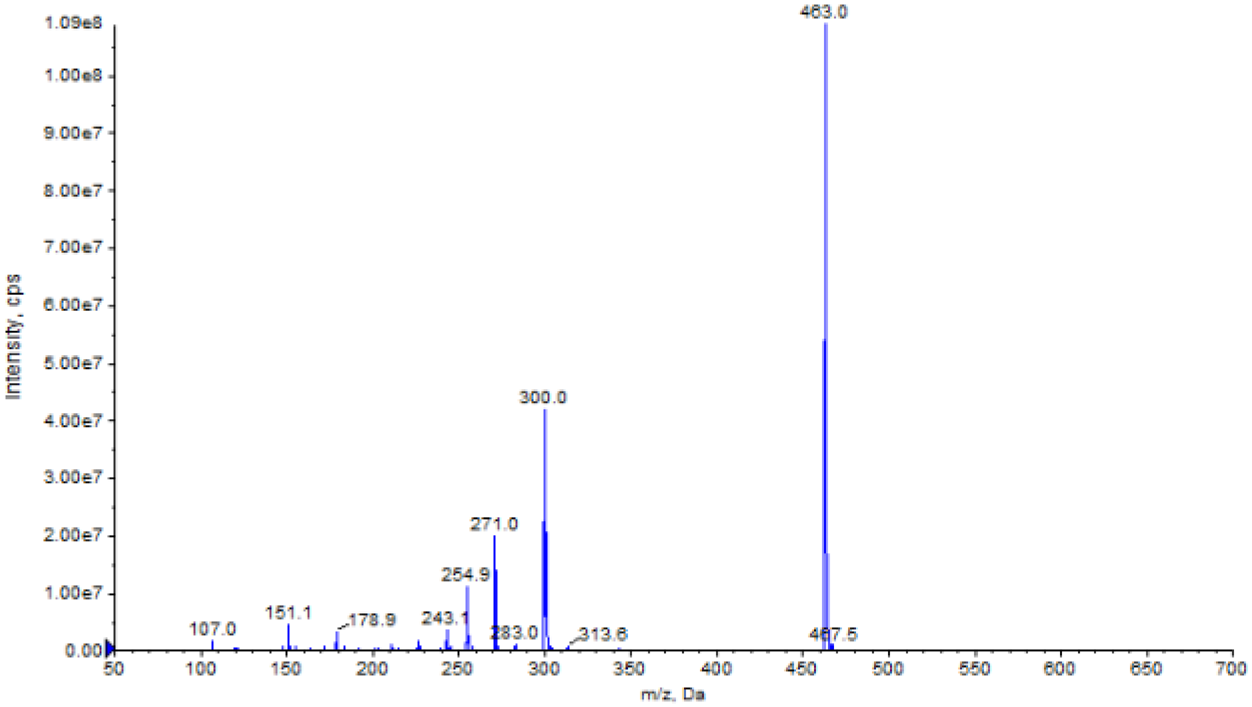

Isoquercitroside

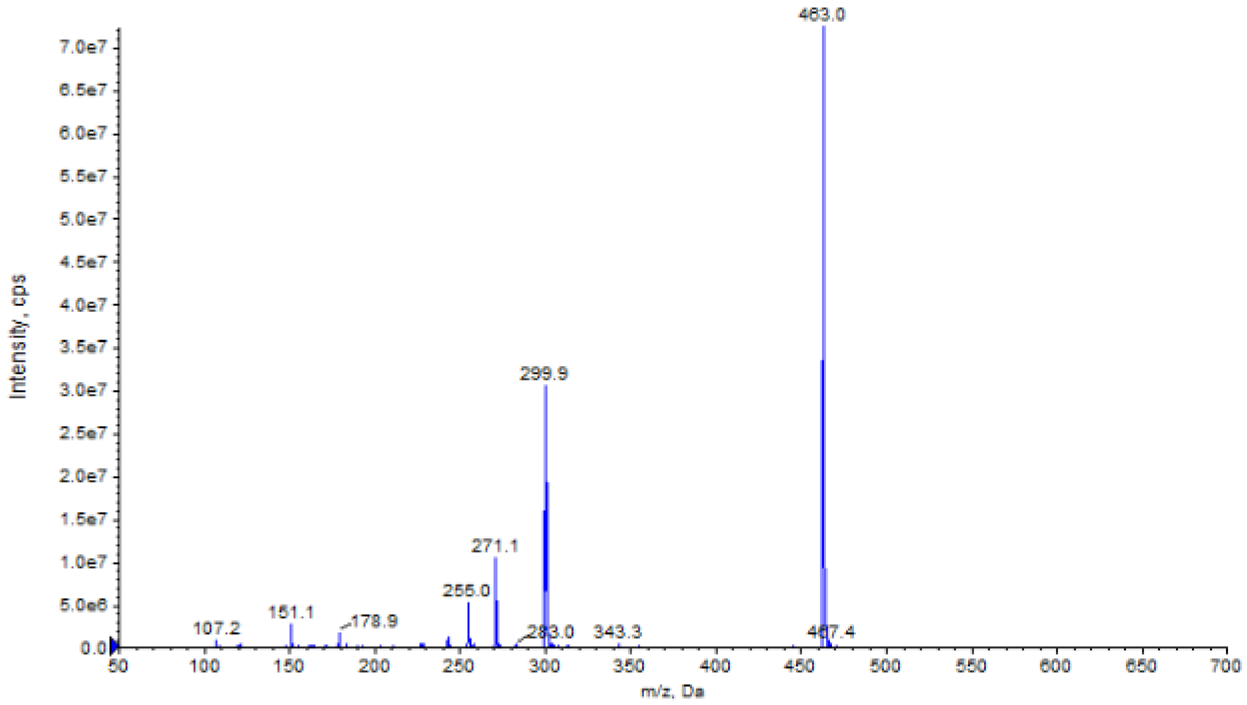

Kaempferol 3-O-arabinoside

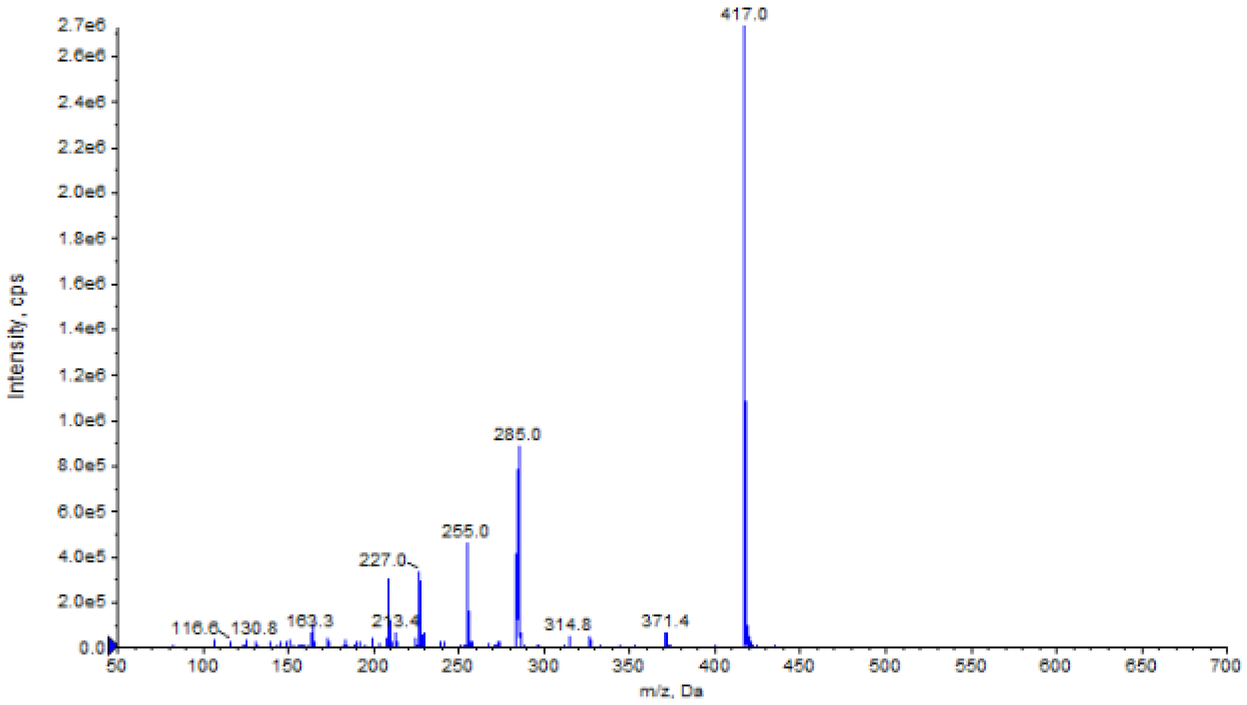

L-Epicatechin

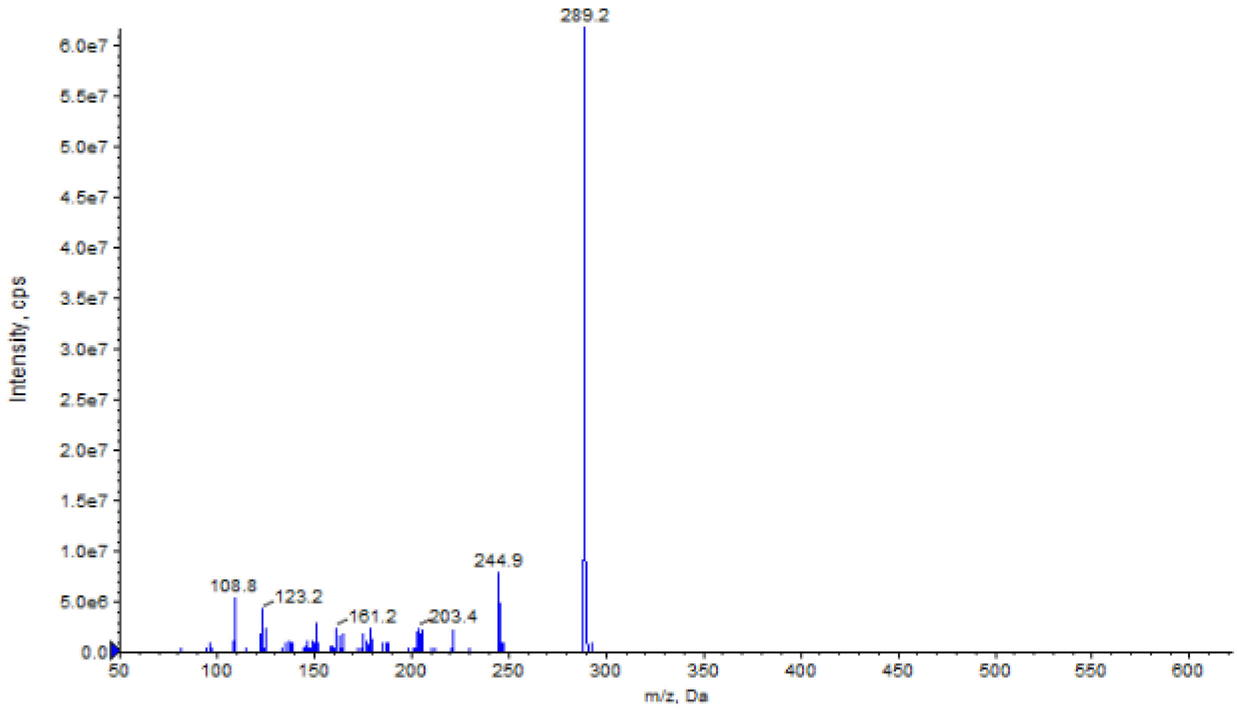

Eriodictyol-7-O-glucoside

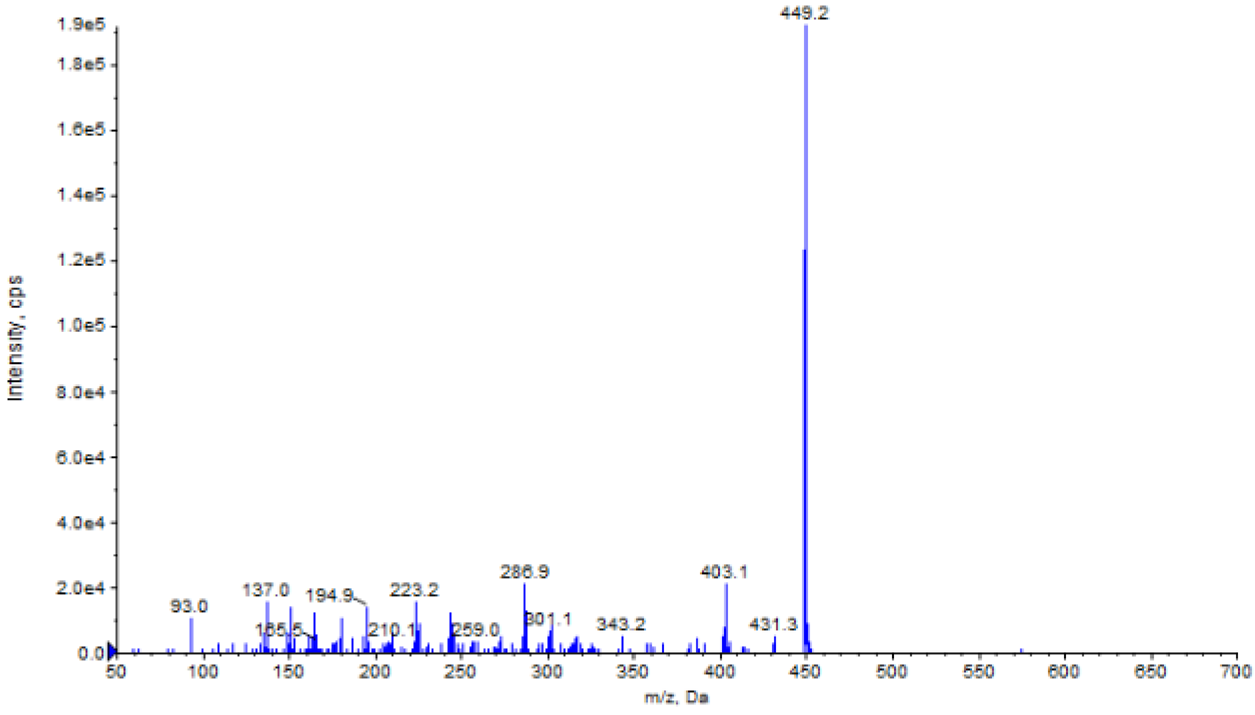

Phloretin xyloglucoside

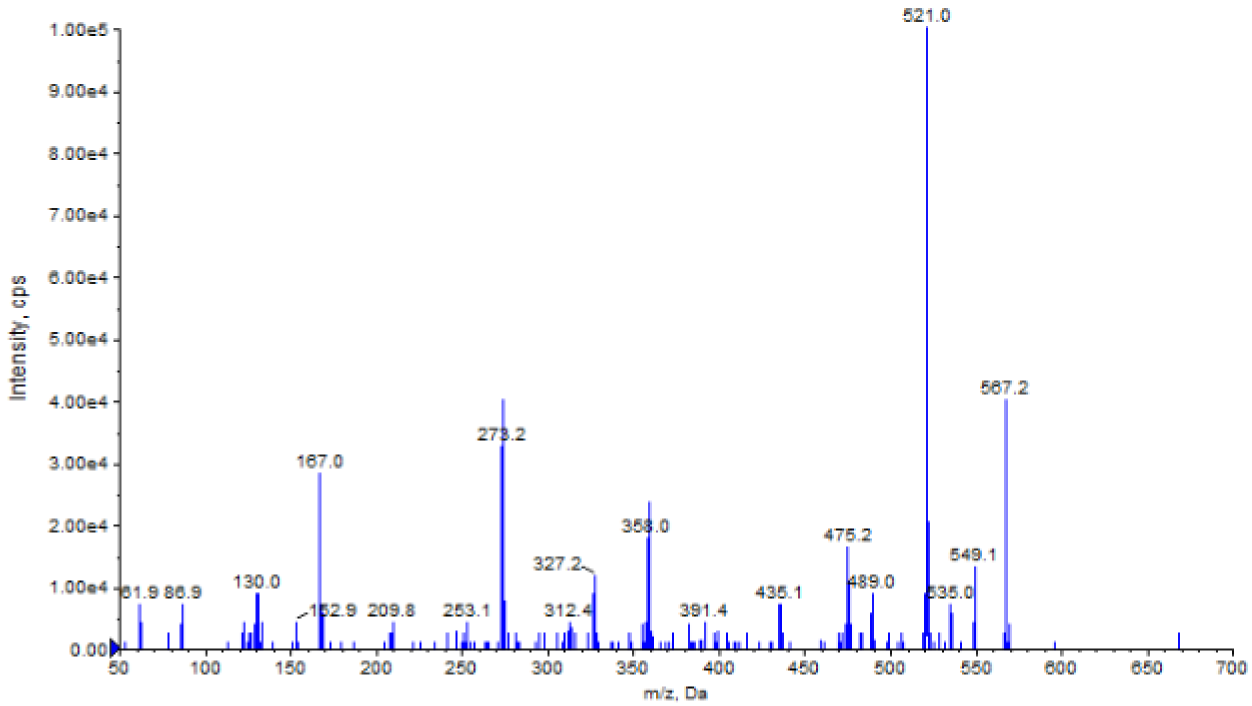

Quercetin

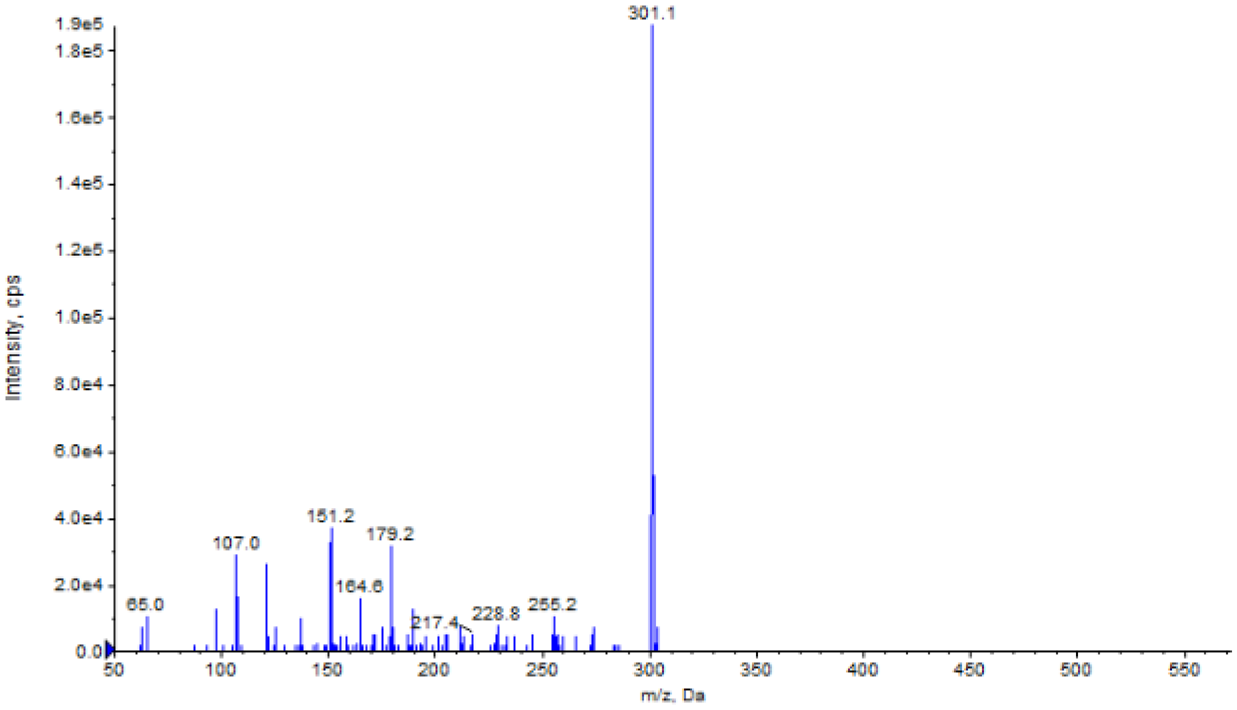

Quercetin 3-O-β-D-xylopyranoside

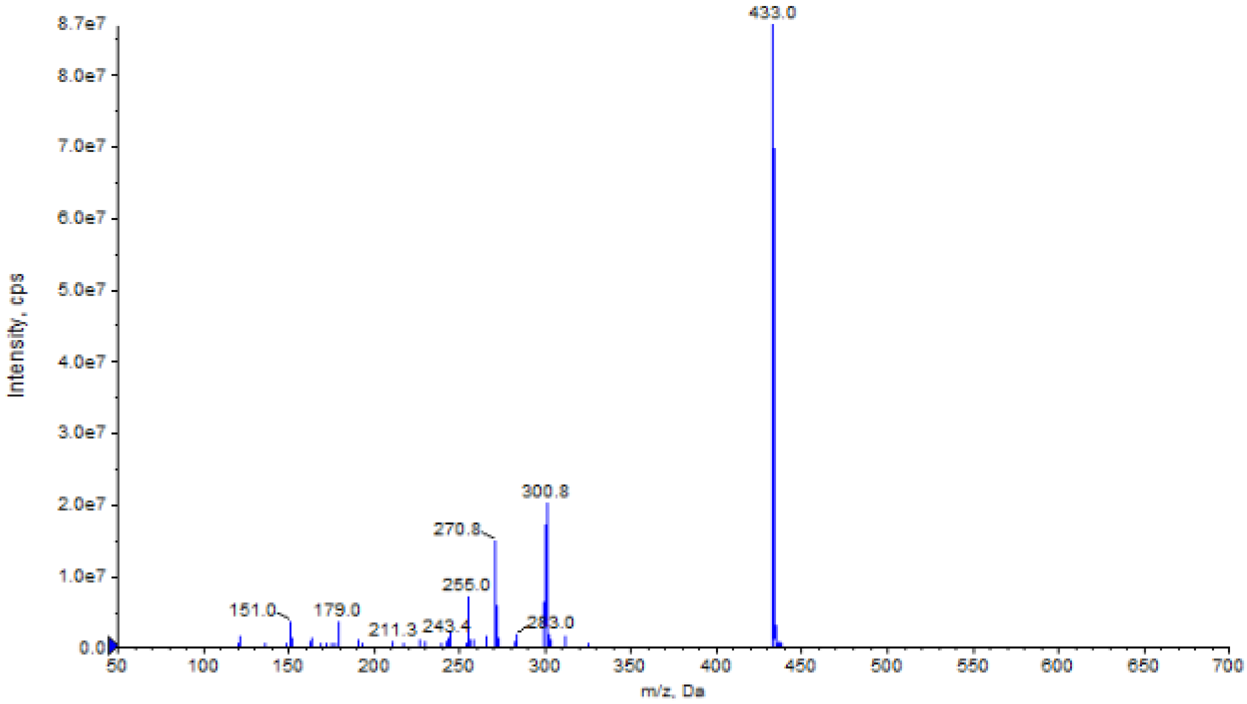

Quercitrin

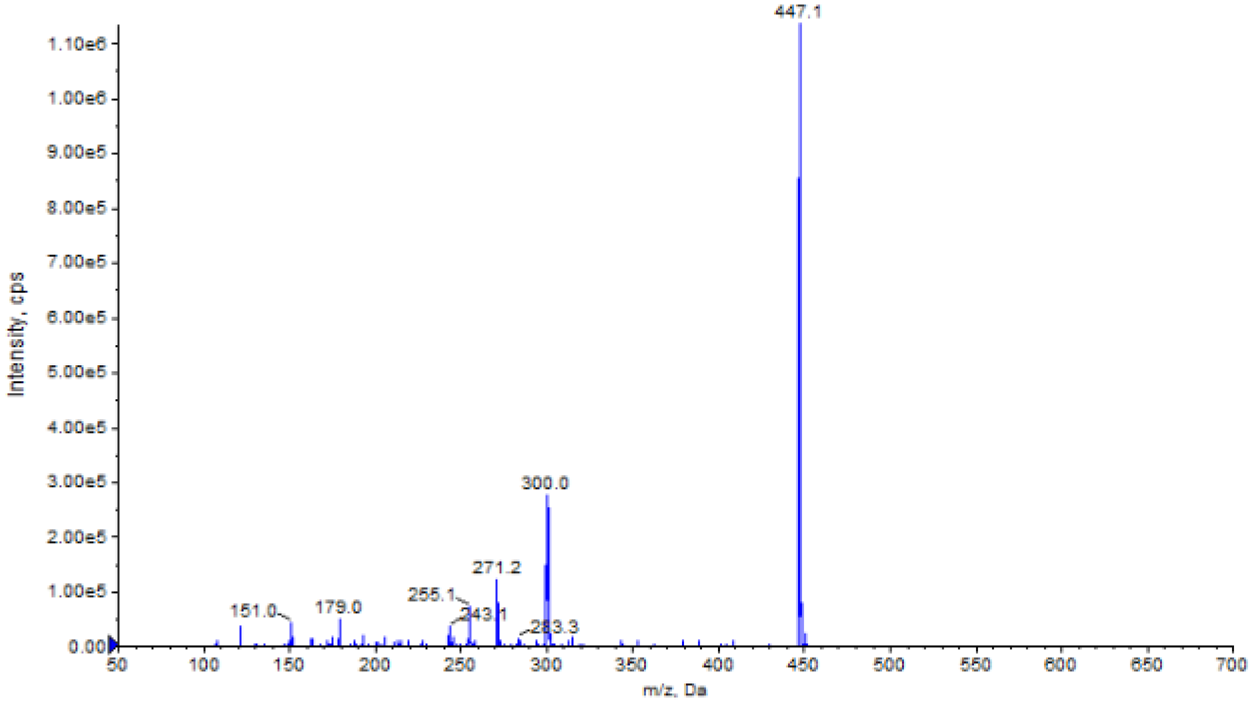

Salicylic acid

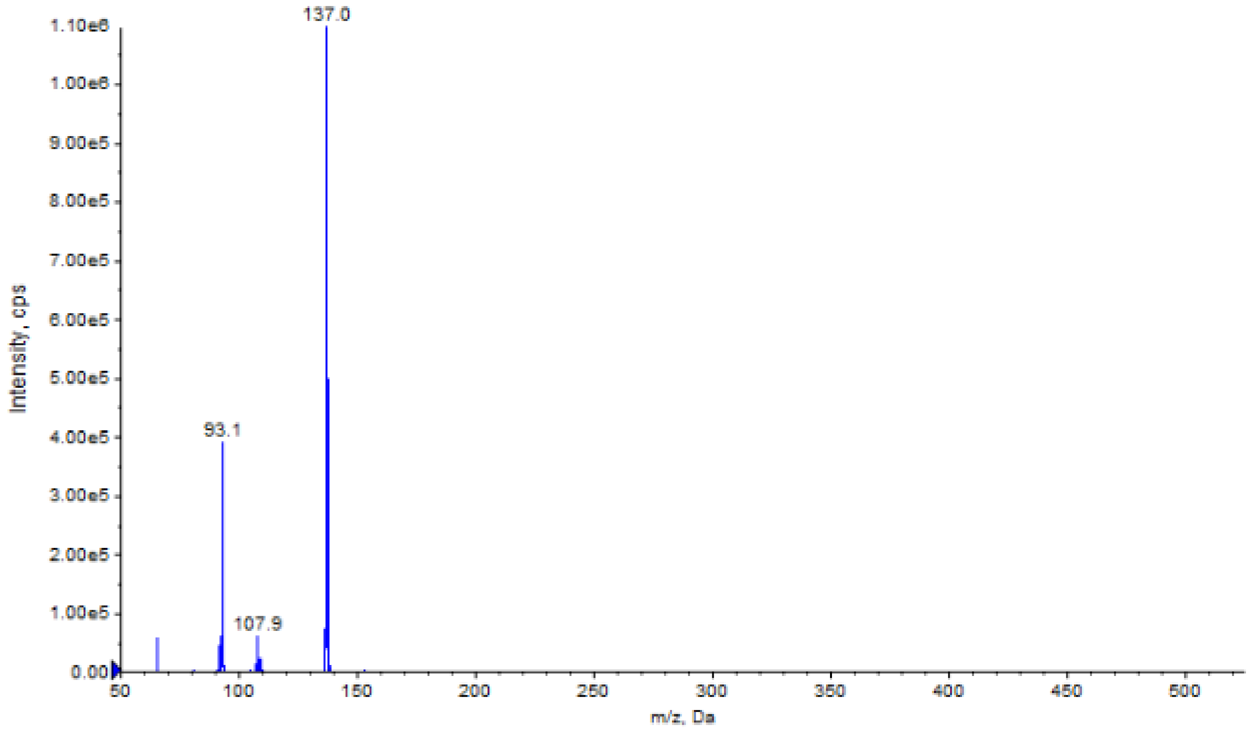

Syringic acid

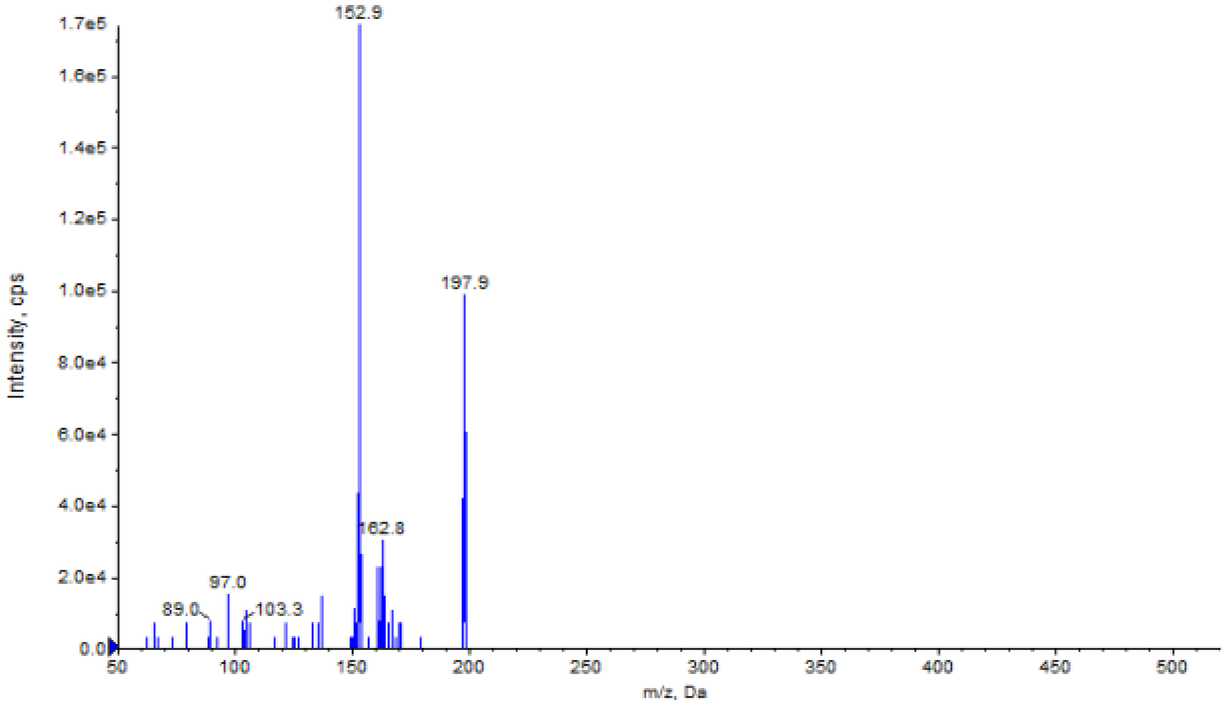

(+)-Catechin

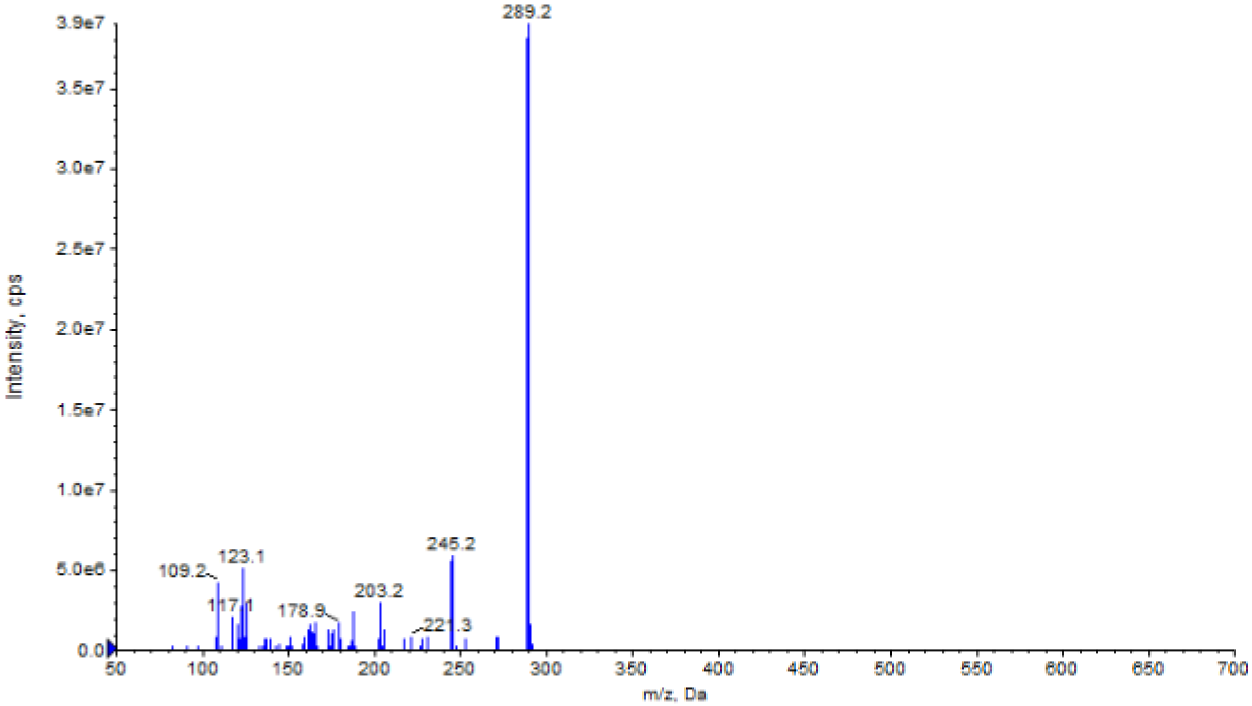

2,5-Dihydroxybenzoic acid

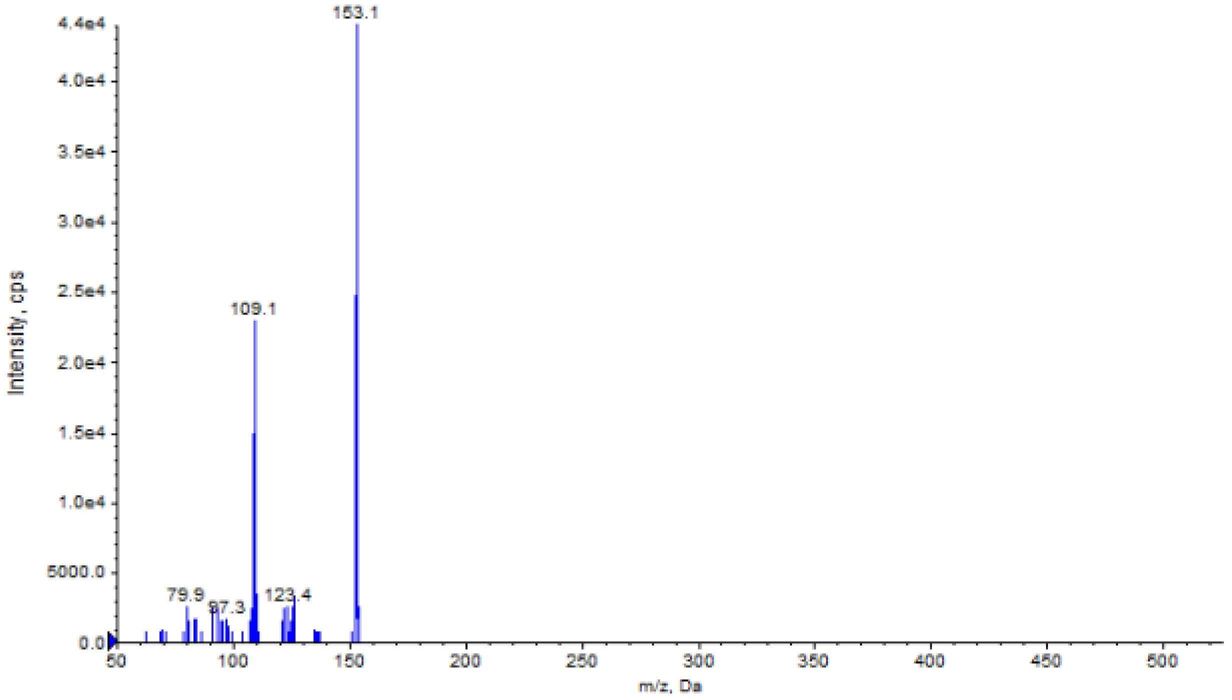

Caffeic acid

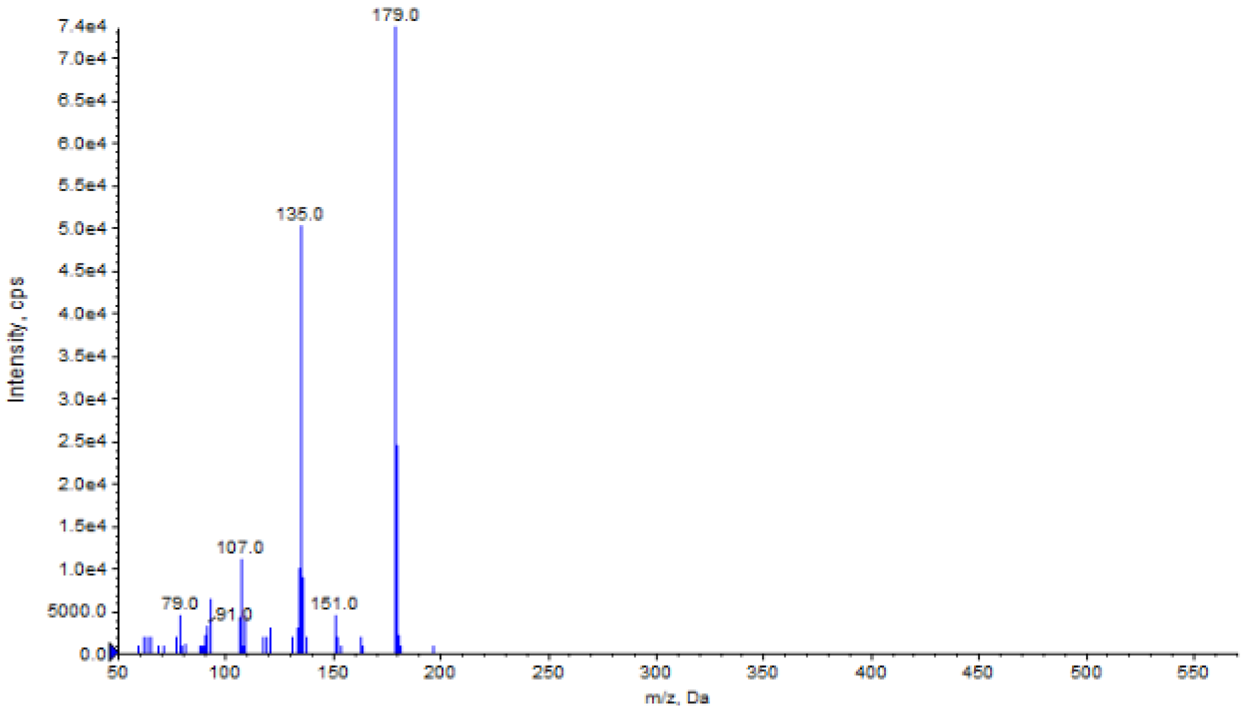

Chlorogenic acid

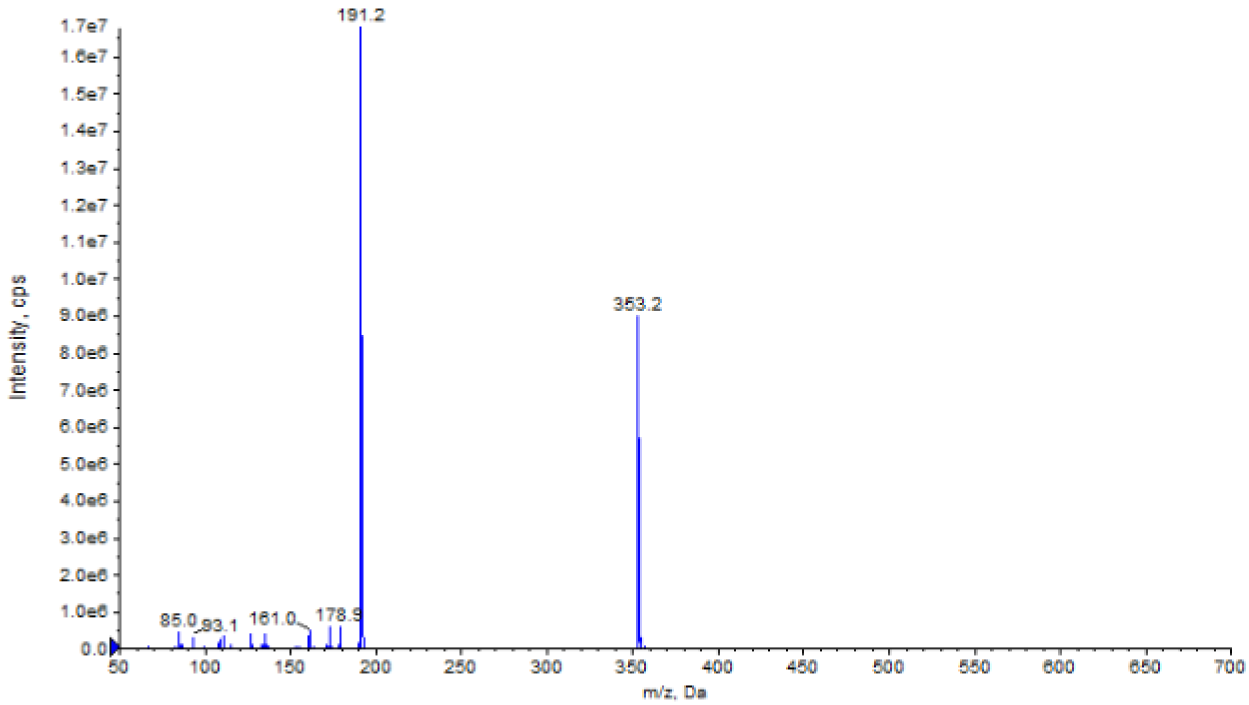

Cinnamic acid

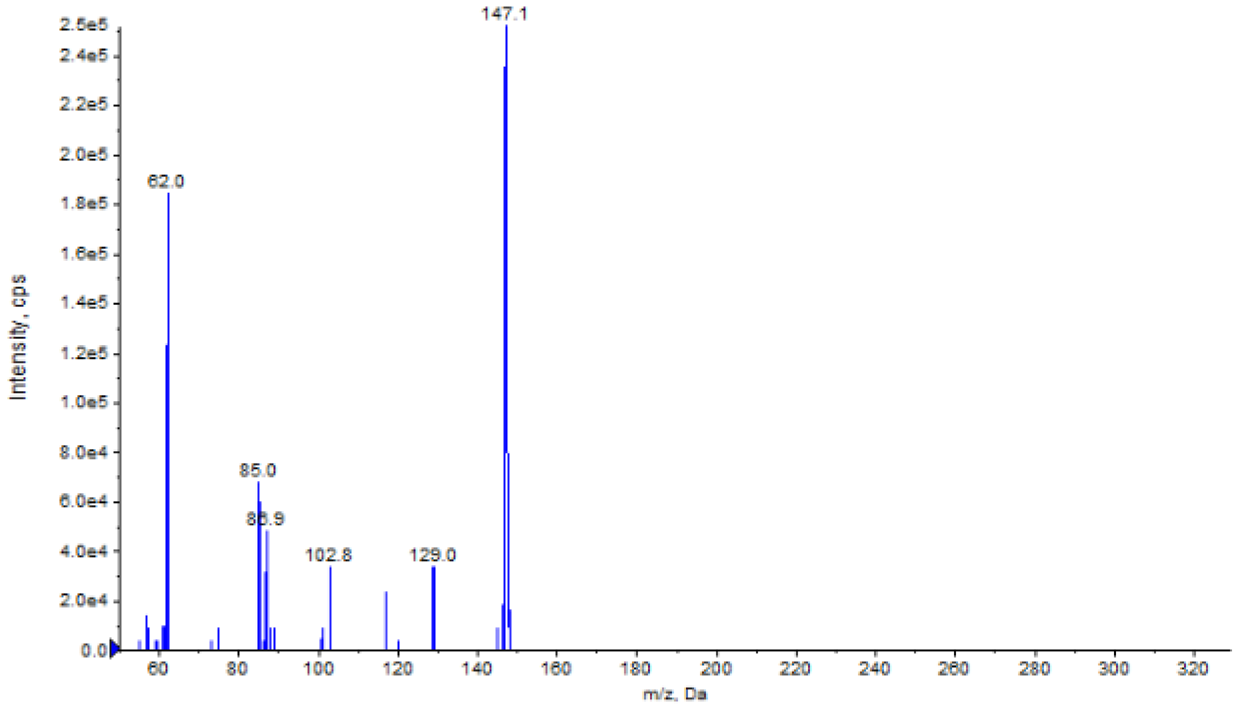

Ferulic acid

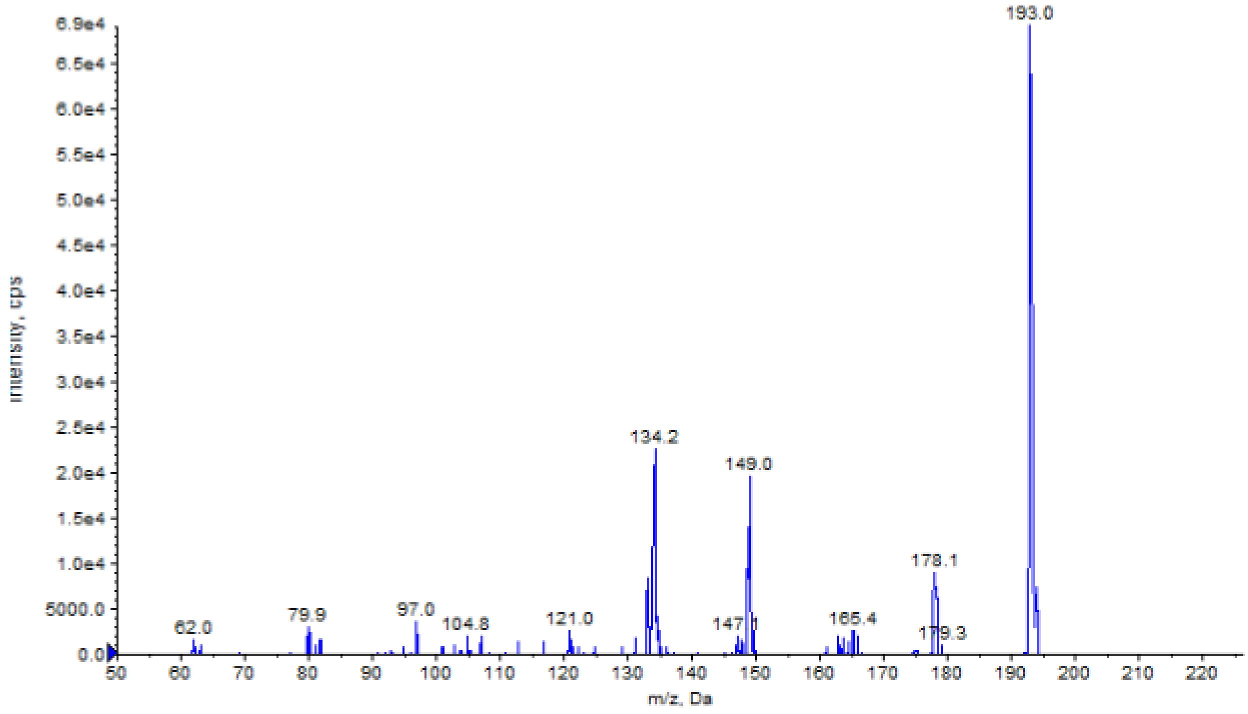

Gallic acid

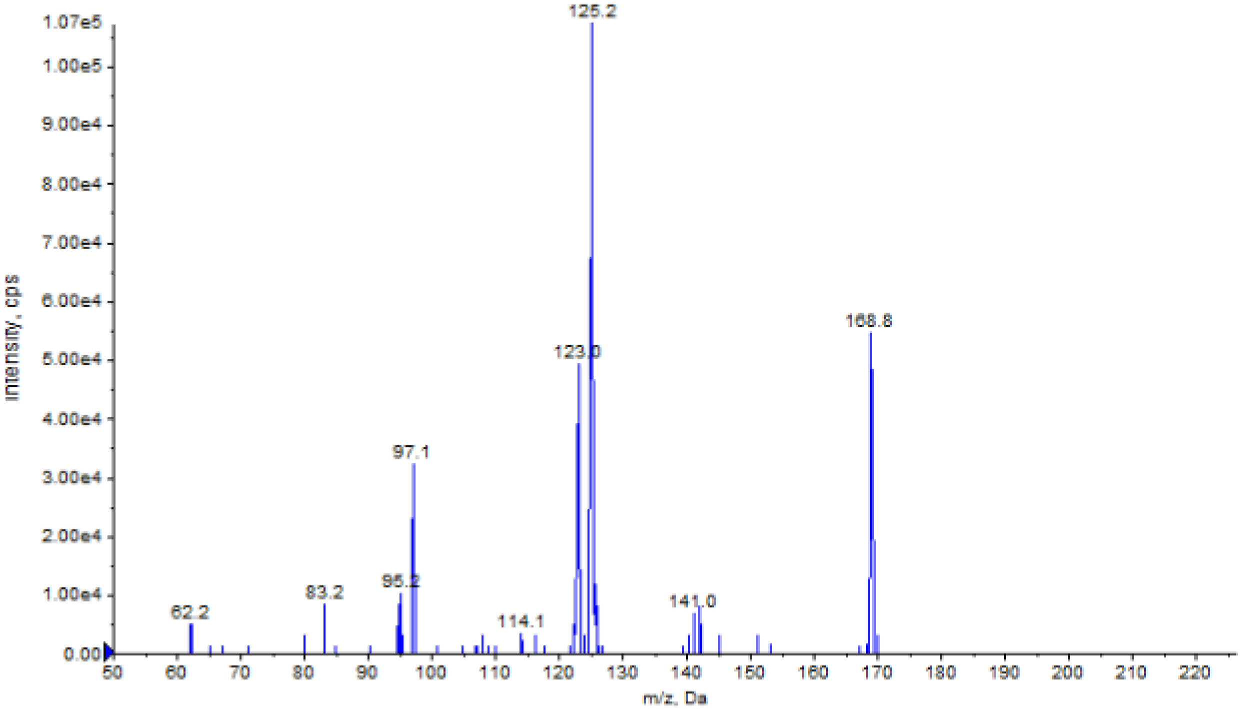

Hyperoside

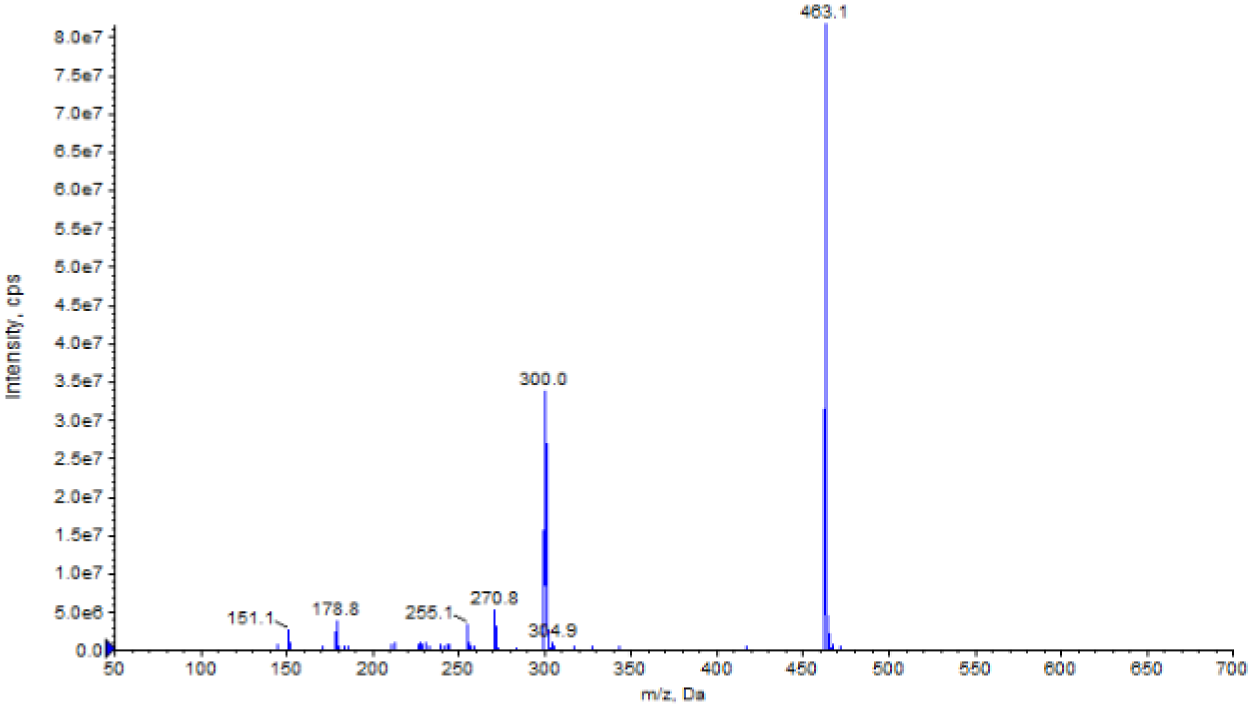

Phlorizin

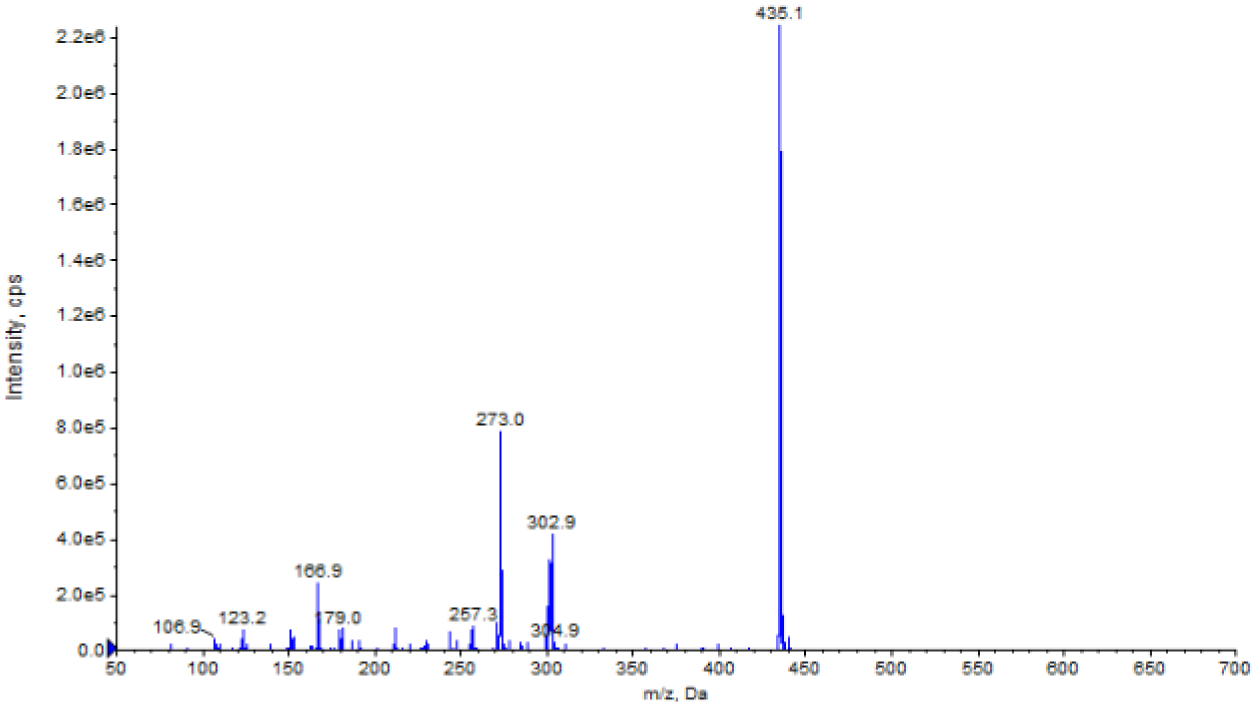

Polydatin

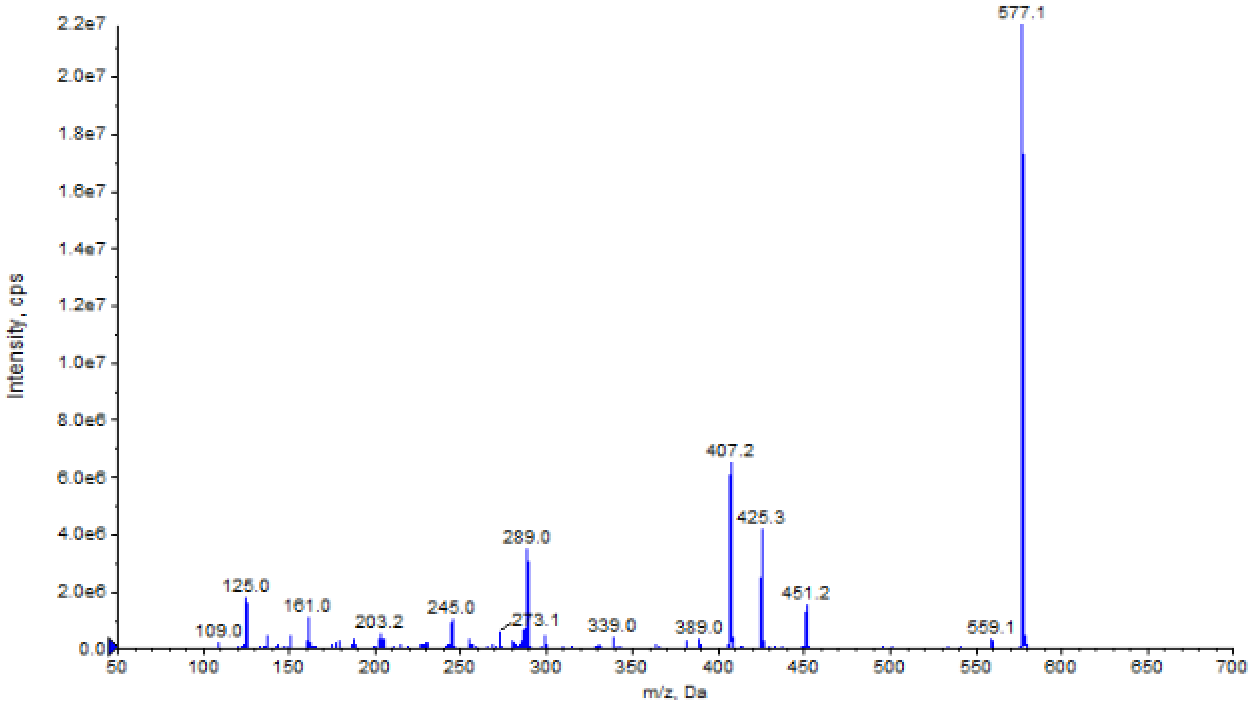

Procyanidin B2

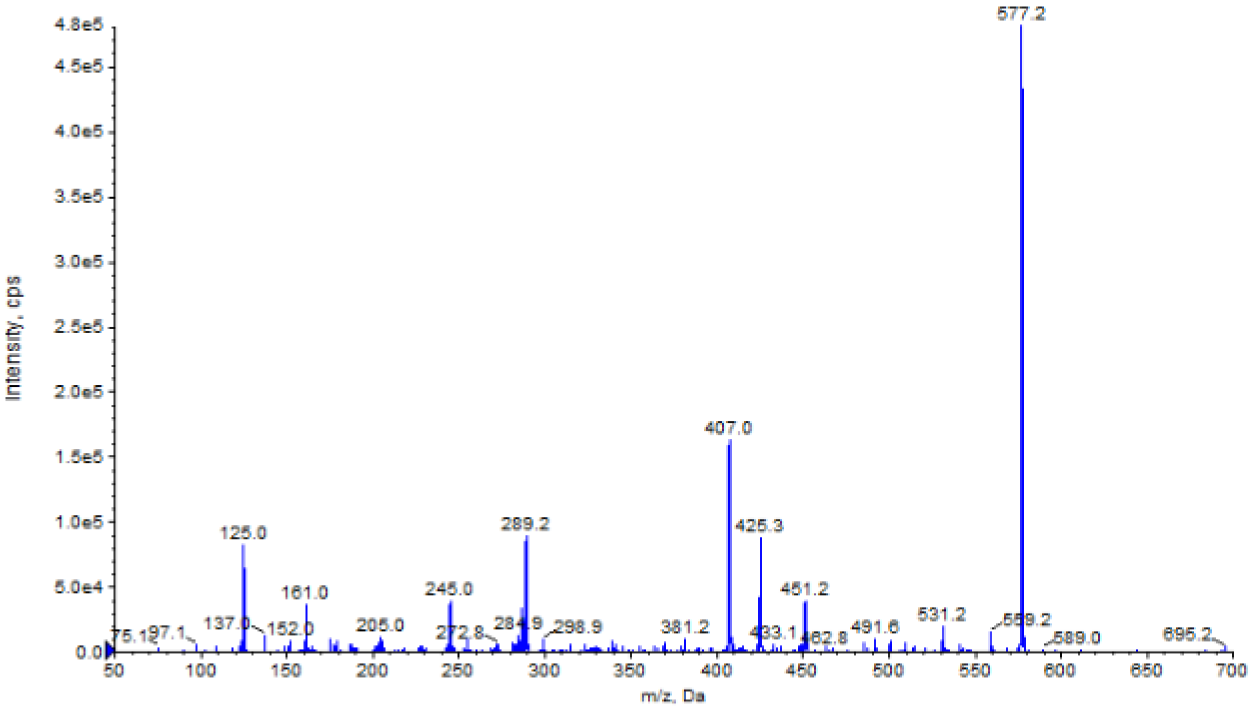

Procyanidin B5

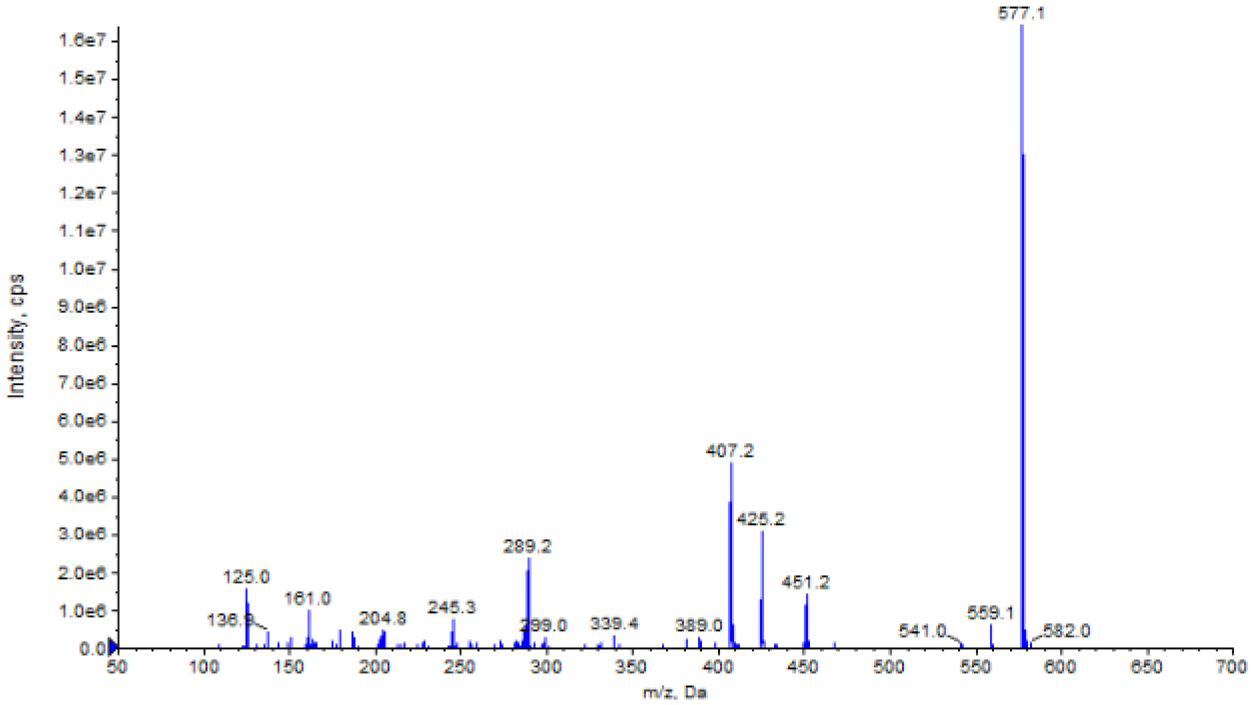

Procyanidin B1

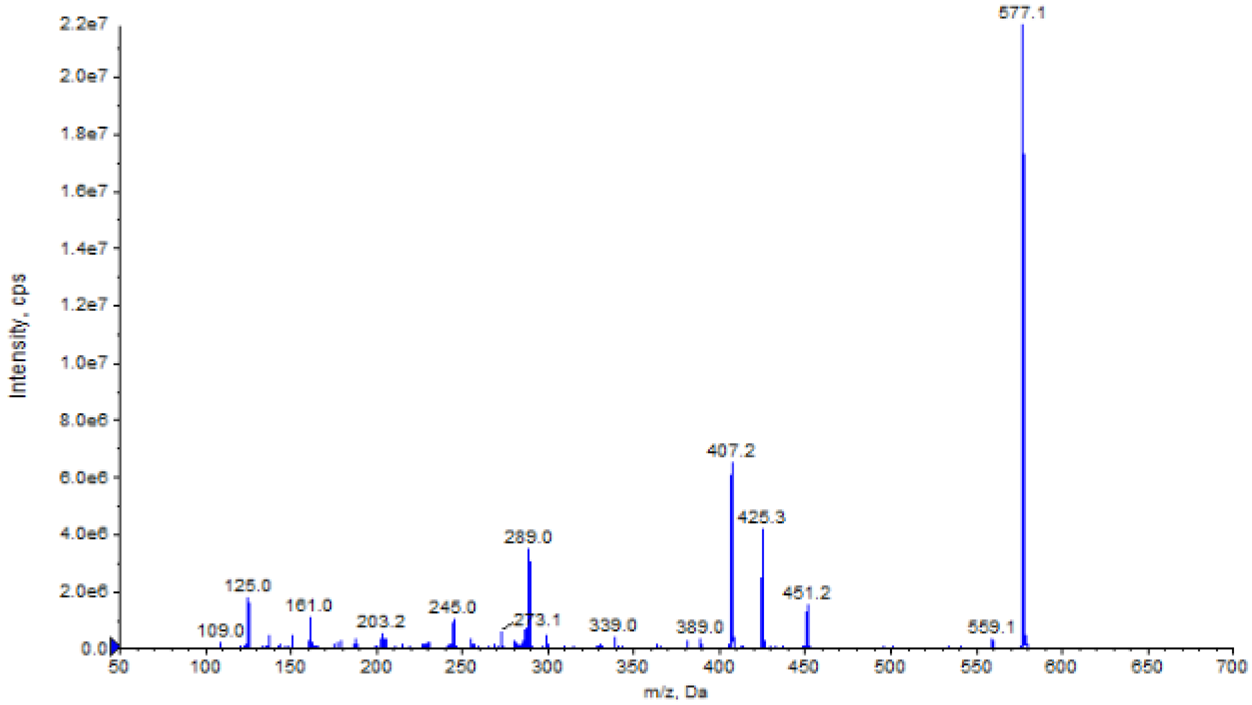

Terephthalic acid

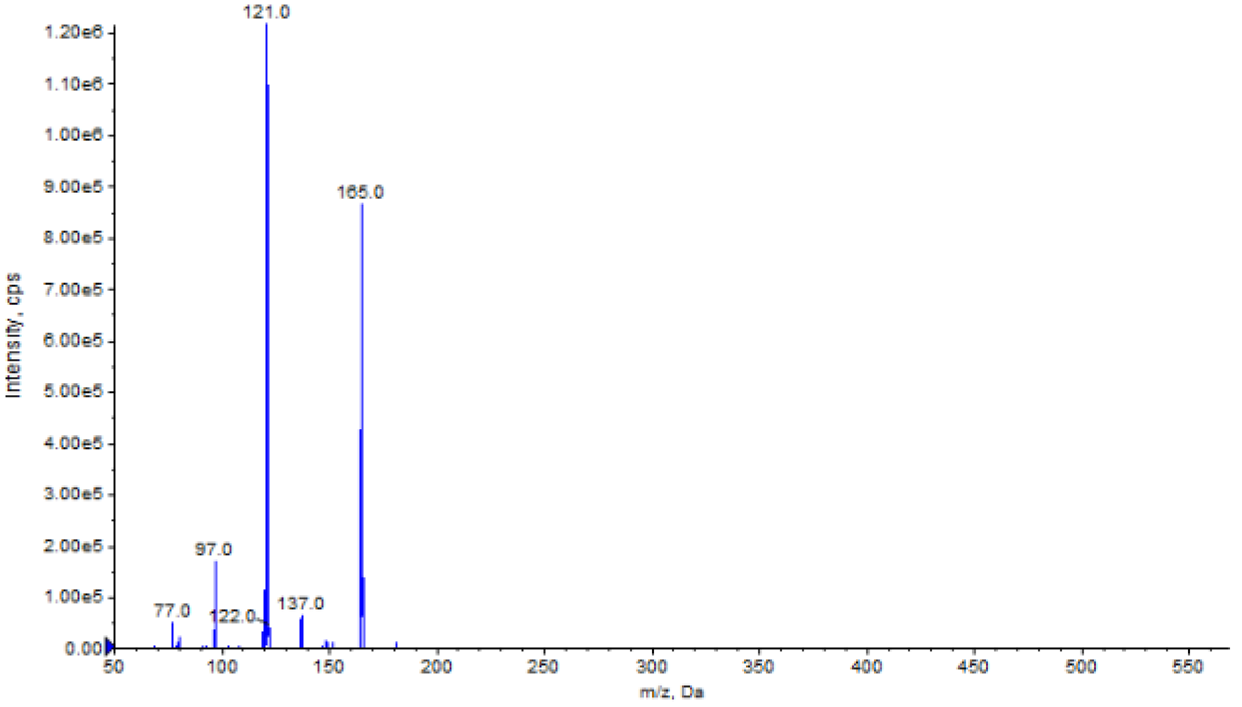

3-Hydroxycinnamic acid

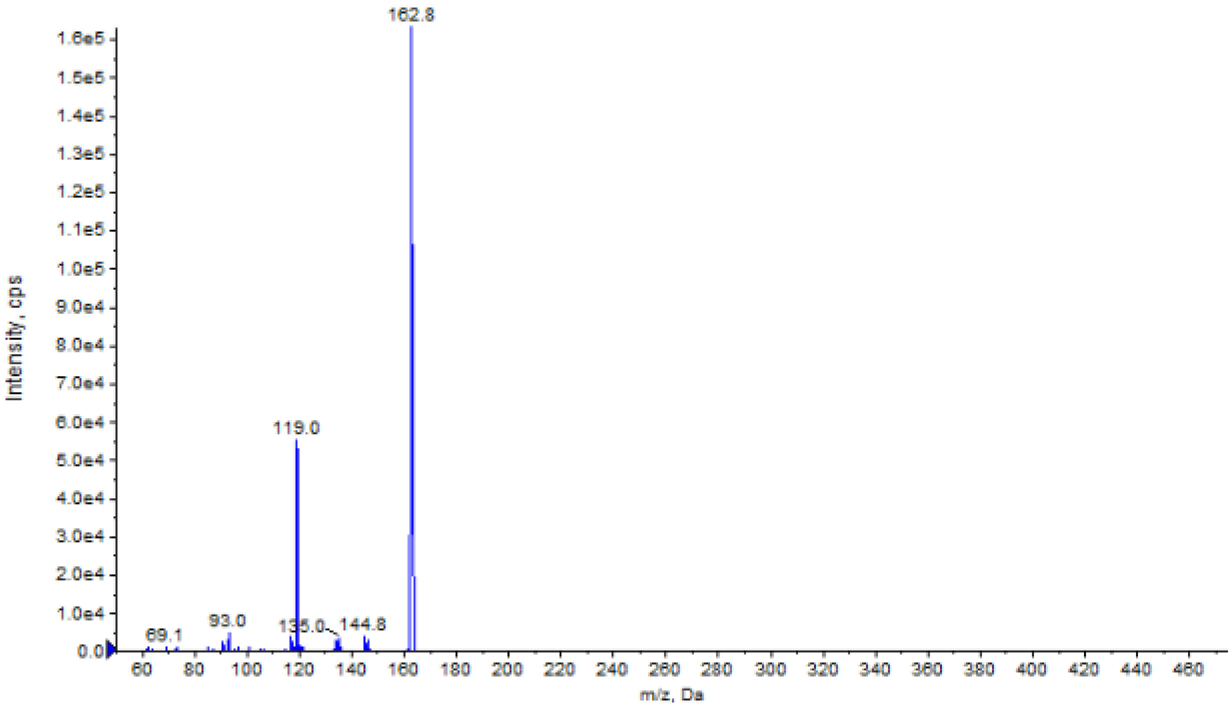

Phloretin

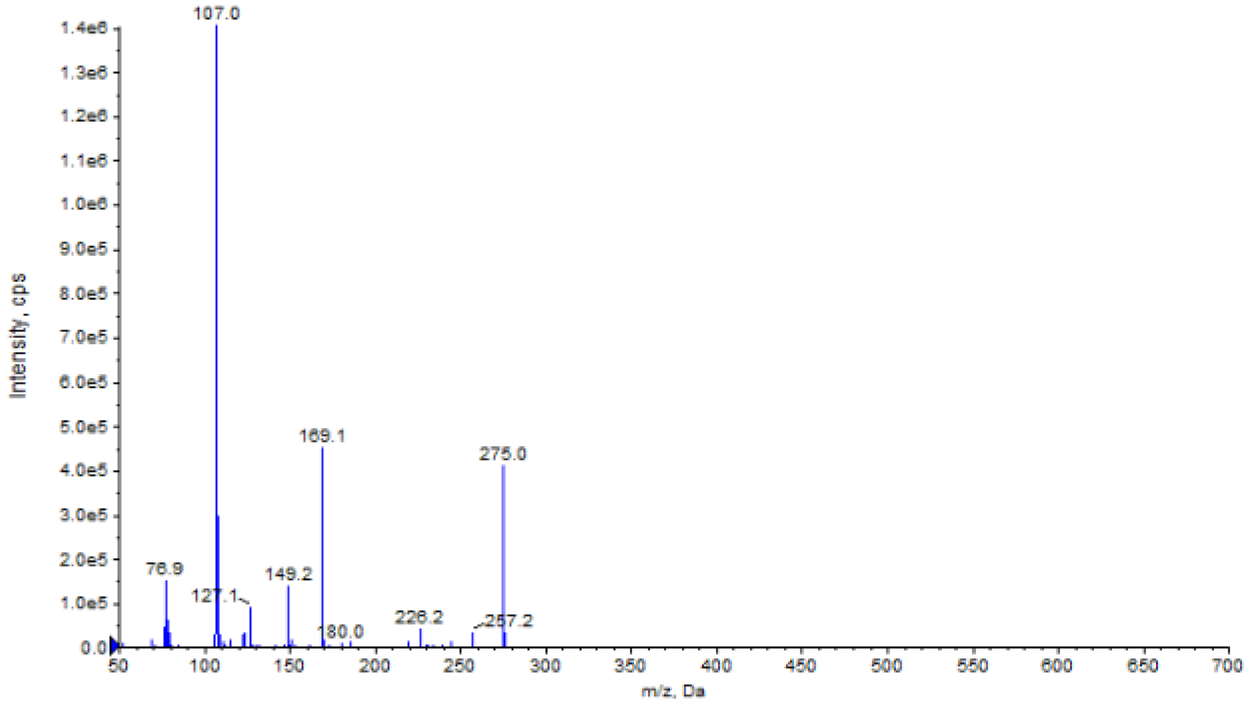

Cosmosiin

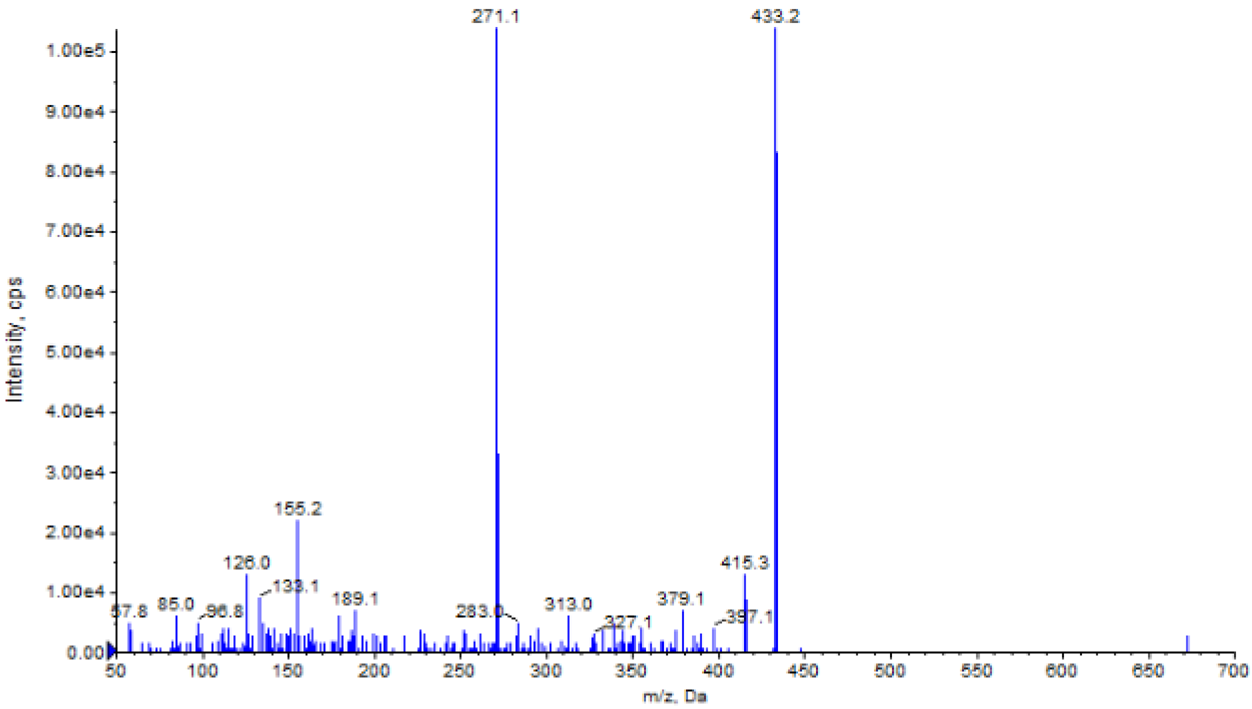

Cyanidin 3-O-glucoside

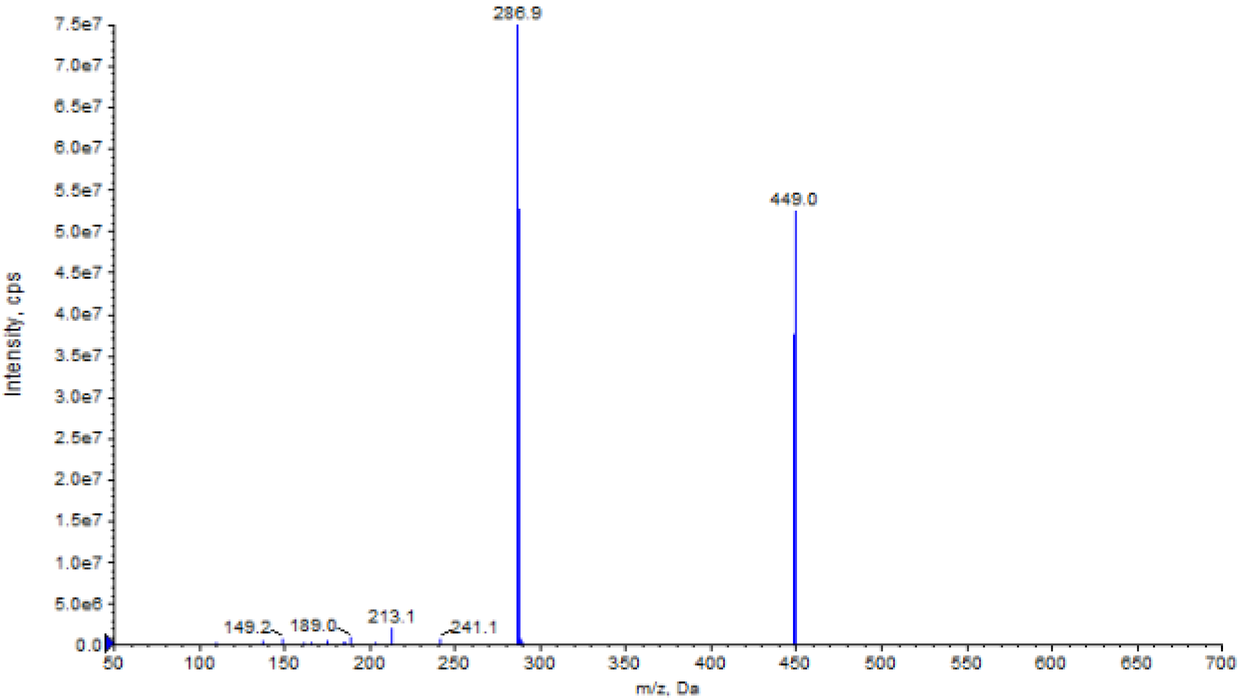

Rutin

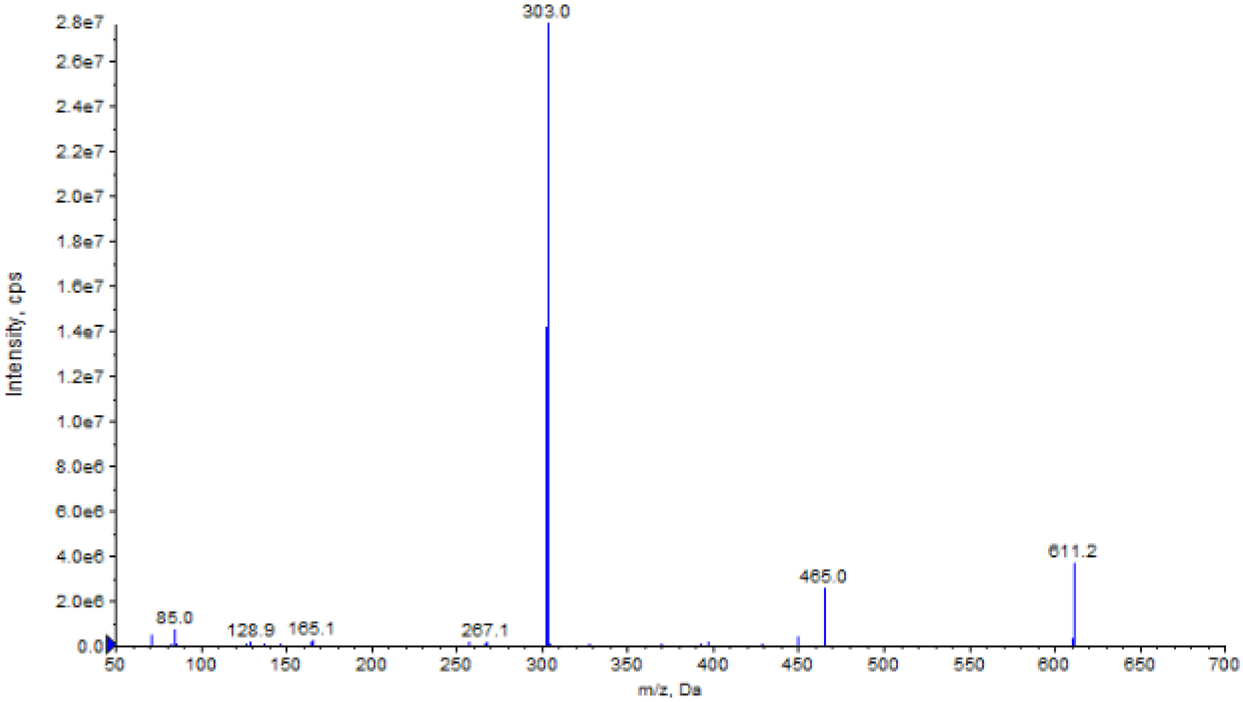

Trilobatin

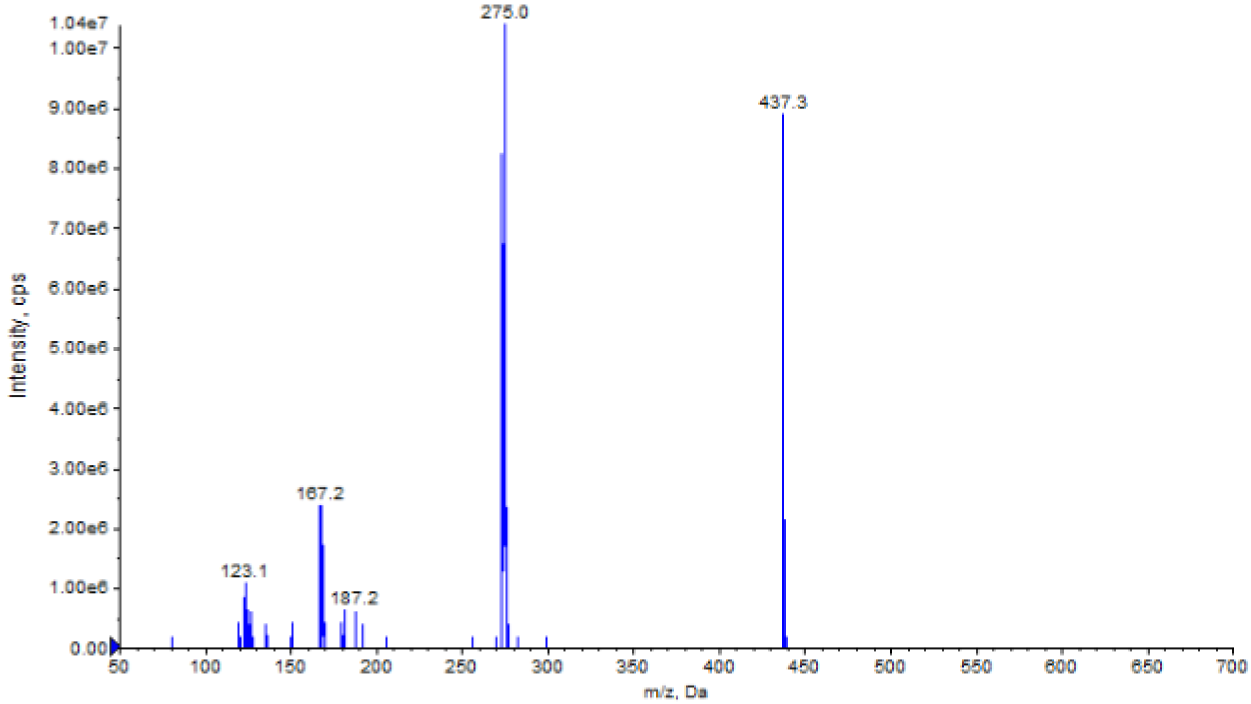

Cyanidin Chloride

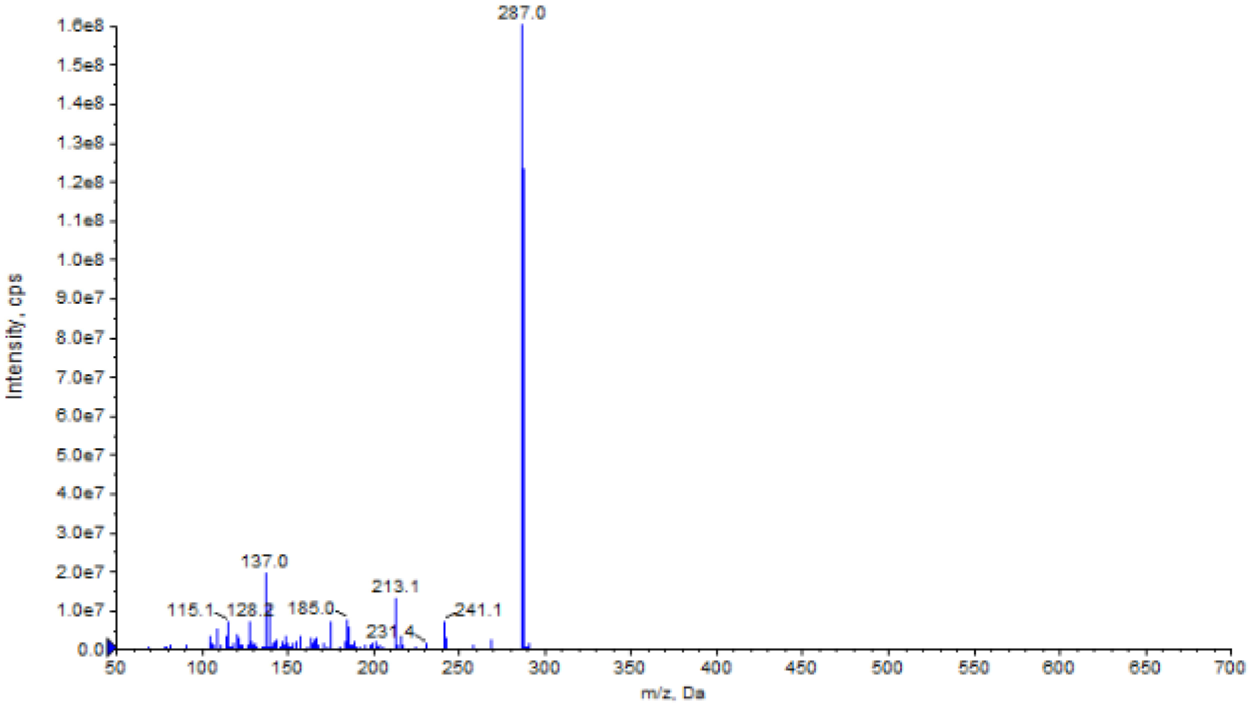

Figure S1. MS2 spectrometry of 39 polyphenol metabolites in apple
