# Supplementary material for: A New Strategy Based on LC-Q TRAP-MS for Determining the Distribution of Polyphenols in Different Apple Varieties
Source: Foods. 2022 Oct 27;11(21):3390. doi: 10.3390/foods11213390 (PMC9657627; doi:10.3390/foods11213390)
Supplement: Supplementary file 1 [file foods-11-03390-s001.zip › Table S1.pdf]

Table S1. Coefficient of variation (CV) of 39 apple polyphenol metabolites.

Compound name	CV of retention time (%)	CV of peak area (%)	Compound name	CV of retention time (%)	CV of peak area (%)
4-P-coumaroylquinic acid	0.06	1.48	Phloretin xyloglucoside	0.17	0.94
(+)-Catechin	0.16	1.59	Phlorizin	0.11	3.76
1-O-Sinapoyl-β-D-glucose	0.13	1.47	Polydatin	0.11	1.22
2,5-Dihydroxybenzoic acid	0.00	1.16	Procyanidin B1	0.06	3.06
3,4-Dihydroxybenzoic acid	0.00	4.92	Procyanidin B5	0.07	4.04
4-caffeoylquinic acid	0.22	4.47	ProcyanidinB2	0.12	0.30
4-Hydroxycinnamic acid	0.10	2.96	Quercetin	0.24	4.32
Avicularin	0.17	0.18	Quercetin 3-O-β-D-xylopyranoside	0.17	2.03
Benzoic acid	0.21	3.70	Quercitrin	0.11	0.39
Caffeic acid	0.06	3.29	Salicylic acid	0.17	4.08
Chlorogenic acid	0.06	4.23	Syringic acid	0.28	3.96
Cinnamic acid	0.53	2.10	Terephthalic acid	0.18	2.34
Ferulic acid	0.94	4.31	3-Hydroxycinnamic acid	0.70	1.94
Gallic acid	0.10	3.43	Phloretin	0.13	3.38
Hyperoside	0.12	1.08	Cosmosiin	0.00	0.68
Isoquercitrin	0.17	0.71	Cyanidin 3-O-glucoside	0.07	0.29
Isoquercitroside	1.23	0.36	Trilobatin	0.11	4.30
Kaempferol 3-O-arabinoside	0.16	1.72	Cyanidin Chloride	0.07	3.35
L-Epicatechin	0.18	2.17	Rutin	0.50	1.44
Eriodictyol-7-O-glucoside	0.42	2.13			
